# Cyclic Glycopeptide Analogs of Endomorphin-1 Provide Highly Effective Antinociception in Male and Female Mice

**DOI:** 10.1021/acsmedchemlett.4c00315

**Published:** 2024-09-17

**Authors:** James E. Zadina, Lajos Z. Szabo, Fahad Al-Obeidi, Xing Zhang, Leticia Ferreira Nakatani, Chidiebere Ogbu, M. Leandro Heien, Torsten Falk, Mitchell J. Bartlett, Robin Polt

**Affiliations:** ^†^Department of Medicine and ^‡^Pharmacology and ^§^Brain Institute, Tulane University School of Medicine, New Orleans, Louisiana 70112, United States; ∥ SE Louisiana Veterans Health Care System, New Orleans, Louisiana 70119, United States; ⊥ Department of Chemistry & Biochemistry, The University of Arizona, Tucson, Arizona 85721, United States; # Department of Neurology, The University of Arizona, Tucson, Arizona 85724, United States; ∇ Departments of Surgery and Neurosurgery, The University of Arizona, Tucson, Arizona 85724, United States

**Keywords:** Opioid, glycopeptide, pain, endomorphin analog

## Abstract

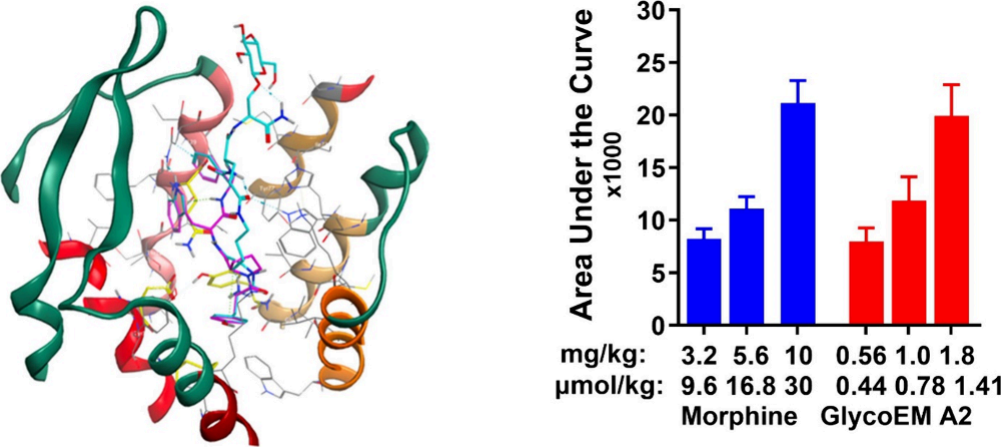

Opioids acting at the mu opioid receptor (MOR) remain the most effective treatment for moderate to severe pain, but their use is limited by serious side effects. We have shown that a cyclized analog of endomorphin-1 provided pain relief comparable to that of morphine with reduction or absence of several side effects, including abuse liability. Glycosylation can promote penetration of cellular barriers. Here we developed cyclic glycopeptide endomorphin (glycoEM) analogs as drug candidates for potent and long-lasting analgesia. The analogs were assessed in receptor binding and functional assays and for blood–brain barrier penetration by microdialysis and MS. Two of the analogs showed MOR selectivity and more potent and longer lasting antinociception than morphine in male and female mice. Comparable antinociception occurred at A2 doses 5-fold lower (20-fold on a molar basis) than morphine doses. The results support further study of the glycoEMs for clinical application.

Opioids acting at the mu opioid receptor (MOR) remain the gold standard for moderate to severe pain relief, but serious side effects, particularly abuse liability, limit their use. Concern with inadequately treated pain, balanced with fear of addiction, has led to pendulums of increased opioid prescribing followed by increased restrictions. According to the CDC the opioid overdose epidemic has grown steadily in roughly three waves: a steady growth since the 1990s with increased opioid prescriptions, sharp increases beginning in 2010 with heroin overdoses and recently with increases in fentanyl overdoses. In 2021,^1^ opioids were involved in 80,411 overdose deaths or 220 deaths/day—over 75% of all drug overdose deaths, and more than occur in auto accidents (95/day^2^). While 20% of these deaths per day are directly tied to prescription drugs, a recent study showed that, of those who began abusing opioids in the 2000s, 75% reported that their first opioid was a prescription drug.^3^ Thus, a compound that provides effective pain relief without addictive properties may help prevent the crucial step of initiation of addiction.

Endomorphins (EMs) are potent and selective natural short peptide agonists for the MOR, the main analgesic target for currently used opioids such as morphine. EM1 shows a distinct binding pattern to MOR.^4^ Shortly after its discovery,^5^ EM1 showed a promising profile of potent analgesia with reduced side effects, including reduced reward^6^ and respiratory depression.^7^ Because the natural peptides are unstable in plasma, medications based on the EMs require chemical modification of the structure (EM analogs). We have described cyclized, d-amino acid-containing EM analogs that were evaluated for (1) metabolic stability for favorable drug properties, (2) highly effective antinociception, and (3) significant reduction of adverse side effects.^8^ After screening of numerous analogs, a MOR-selective lead (ZH853) emerged with a highly favorable analgesia/side-effect profile. Doses of ZH853 that produced durations of antinociception equal to, or significantly longer than, that of morphine, exhibited significantly reduced side effects compared to morphine. The analog showed reduced a) respiratory depression, b) impairment of motor coordination, c) tolerance and hyperalgesia, d) glial activation and p38/CGRP/P2X7 receptor signaling, and e) reward/abuse potential in both conditioned place preference (CPP) and self-administration (SA) tests.^8^ ZH853 also showed potential as a treatment for opioid use disorder.^9^

Some degree of blood-brain barrier (BBB) penetration of ZH853 is supported by the demonstration that central administration of the antagonist naloxone-methiodide inhibited antinociception after peripheral (i.v.) administration of ZH853 in rats.^8^ Additionally, this cyclic peptide attenuated thermal nociception in the hot plate test in male and female mice, a centrally mediated pain response. However, additional studies indicate that its BBB penetration is much lower than morphine. Studies using both peripheral and intrathecal administration^8,10,11^ in multiple pain models showed much greater potency of ZH853 relative to morphine after central administration (average ∼62-fold more potent) while potencies were similar after peripheral administration.

This suggests differential kinetics or reduced BBB penetration relative to morphine, but greater pharmacodynamic effects of the cyclic EM analog. Thus, additional modifications of endomorphin were introduced to improve the penetration of cellular barriers, lower the required doses for analgesia and limit peripherally mediated side effects.

Glycosylation is a major natural modification that occurs on over half the mammalian proteins.^12^ Glycosylation of a peptide has been shown to extend serum stability and increase transport across cellular barriers, principally at the BBB.^13^ Adsorptive transcytosis is driven by amphipathic interactions of the glycopeptides with biological membranes as well as with their cognate GPCR receptors.^14^ Several studies support the concept that glycosylation of peptides can increase penetration of the BBB.^15−22^ Increased oral availability has also been demonstrated for one of these analogs.^22^ In this study we combined glycosylation with cyclization, which can stabilize active conformations and extend peptide lifetimes.^13^ We therefore synthesized seven cyclic endomorphin-1 glycosides (A1–A7) and an unglycosylated control peptide (A8) (Figure 1; see Table 2 below) and tested them for receptor binding and antinociception in both tail flick and hot plate after peripheral (s.c.) administration in mice. In silico modeling and BBB penetration were characterized for glycoside A1.^20,23^

**Figure 1:**
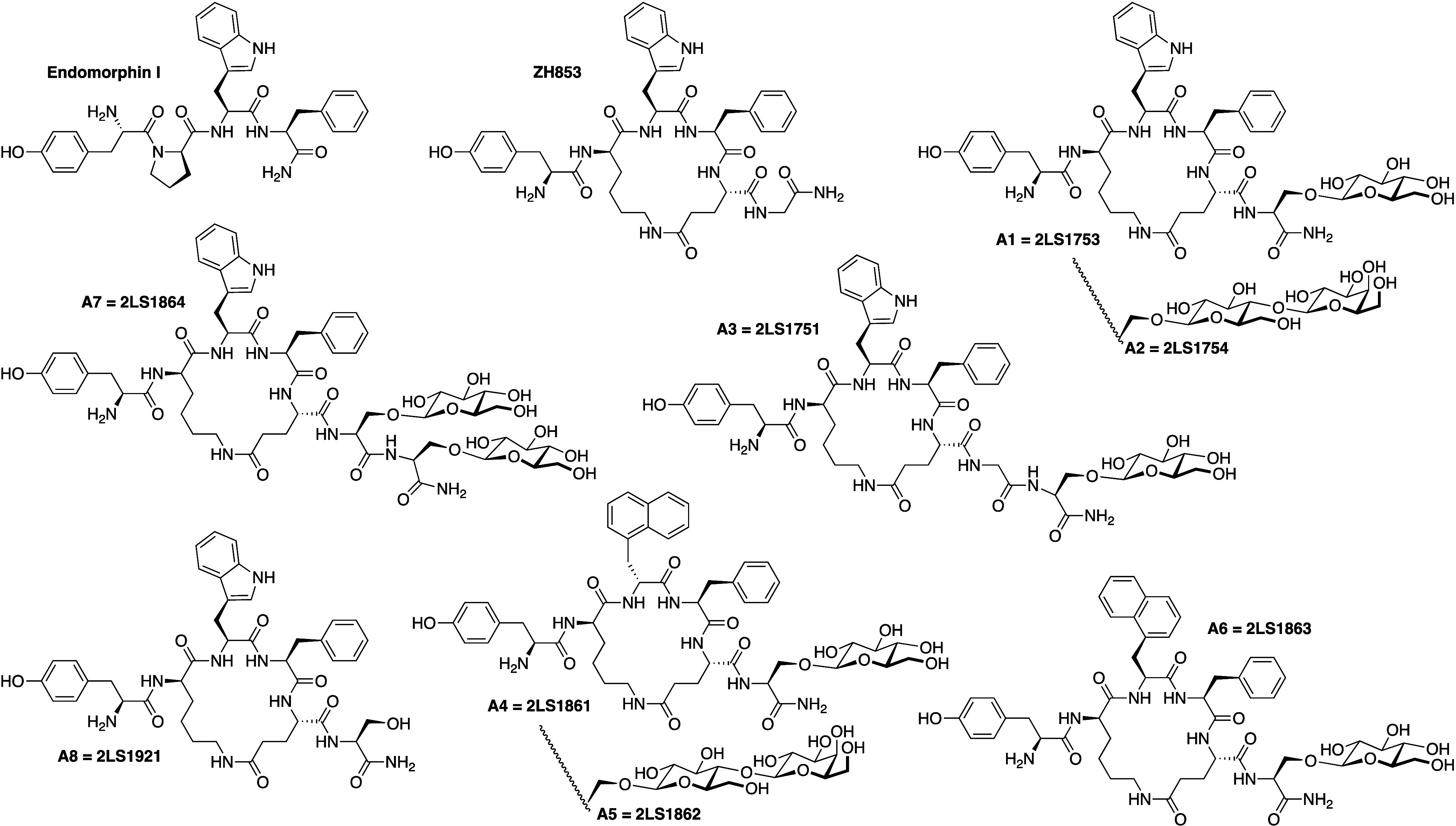
Structures of seven c-terminal glycosylated analogs of endomorphin (A1–A7) tested in this study and compared to reference compounds ZH853, A8 and morphine.

## Chemicals/Glycopeptides

Morphine was generously supplied by the NIDA Drug Supply Program. The glycopeptides were produced using Fmoc-based SPPS (Solid-Phase Peptide Synthesis), reversed phase HPLC purification and HRMS characterization, as described in the Supporting Information.

## In Silico Modeling

Comparison of the lactam conformations, e.g. glucoside A1, with the native EM1 structure, as determined by CryoEM,^4^ showed that the pharmacophores were closely aligned in the binding site (Figure S2).

## Blood-Brain Barrier Penetration.

BBB penetration and pharmacokinetics of the A1 glucoside and ZH853 were determined *in vivo* as described^20,23^ and illustrated in the Supporting Information. Following intravenous bolus injection, AUC, Cmax, half-life, and Tmax were determined in plasma and CSF dialysate, and the CSF/plasma ratios for AUC of the analogs were compared (Table 1). The AUC CSF/plasma ratio was 4.76-fold greater for A1 than ZH853, supporting the concept of improved BBB penetration by glycosylation.

**Table 1:** Pharmacokinetics and BBB Penetration

	AUC (μM*min)			Cmax (μM)		Half-life (min)		Tmax (min)	
Analog	Plasma	Brain	CSF/Plasma	Plasma	Brain	Plasma	Brain	Plasma	Brain
A1	157 ± 37	141 ± 69	0.898	16 ± 7	11 ± 7	26 ± 6	17 ± 8	1	5
ZH853	110 ± 37	21 ± 8	0.189	17 ± 3	2 ± 0.2	16 ± 2	6 ± 0.7	2	28
A1/ZH853			4.76						

## Receptor Binding and cAMP Profiles

*K*_i_ determinations at the 3 canonical opioid receptors, MOR, DOR. and KOR. were generously performed by the NIMH Psychoactive Drug Screening Program (PDSP), directed by Bryan L. Roth at the University of North Carolina at Chapel Hill and Project Officer Jamie Driscoll at NIMH, Bethesda. MD.

Receptor binding affinity is shown in Table 2 for mu, delta and kappa (μ, δ, κ, MOR, DOR, KOR) opioid receptors. The reference cyclic peptide ZH853, with a subnanomolar *K*_i_, showed the highest MOR affinity followed by the unglycosylated-Ser analog (A8). Glycosylation shifted the affinity ∼5-fold for analogs A1, A2, A6 and A7. Substitution of Trp3 with d-Nal (A4, A5) further reduced MOR binding an additional 11-fold and eliminated delta and kappa binding entirely. l-Nal substitution (A6), however, did not further reduce MOR affinity but increased KOR affinity, resulting in the least selective compound. By contrast, the double glycoside (A7) reduced DOR and KOR affinities, resulting in the most selective glycopeptide. Unexpectedly, insertion of a Gly spacer before the Ser glucoside (A3) reduced MOR and increased KOR affinity, resulting in the lowest MOR affinity and the only analog with higher KOR than DOR affinity. Binding studies also showed that the analogs did not bind significantly (>10 μM) to 28 key off-target sites: 8 Adrenergic, 7 Serotonin, 4 Dopamine, 3 Histamine, 2 Muscarinic, 2 Benzodiazapine, GABAa, and Sigma-2 receptors, as well as the Dopamine Transporter (DAT).

**Table 2:** Compound Properties and Receptor Binding and Selectivity^a^

									*K*_i_ (nM)			Selectivity	
	FW·TFA	MW	AA1	AA2	AA3	AA4	AA5	AA6 (and AA7)	MOR (μ)	DOR (δ)	KOR (κ)	δ/μ	κ/μ
ZH	868.9 (Ac)	809.9	Tyr	d-Lys	Trp	Phe	Glu	Gly-CONH_2_	0.61 ± 0.2	385 ± 61	1435 ± 306	631	2353
A1	1116.4	1002	Tyr	d-Lys	Trp	Phe	Glu	Ser(β-d-Glc)-CONH_2_	3.56 ± 1.1	688 ± 168	1280 ± 213	193	359
A2	1278.2	1164.2	Tyr	d-Lys	Trp	Phe	Glu	Ser(β-Lact)-CONH_2_	3.37 ± 1.2	1016 ± 370	10000	301	2967
A3	1173.1	1059.1	Tyr	d-Lys	Trp	Phe	Glu	Gly-Ser(β-d-Glc)-CONH_2_	162.08 ± 6.0	10000	659 ± 146	62	4
A4	1127.1	1013.1	Tyr	d-Lys	1-d-Nal	Phe	Glu	Ser(β-d-Glc)-CONH_2_	45.23 ± 9.2	10000	10000	221	221
A5	1289.3	1175.3	Tyr	d-Lys	1-d-Nal	Phe	Glu	Ser(β-Lact)-CONH_2_	67.16 ± 6.9	10000	10000	149	149
A6	1127.1	1013.1	Tyr	d-Lys	l-Nal	Phe	Glu	Ser(β-d-Glc)-CONH_2_	2.61 ± 1.0	334 ± 75	370 ± 75	128	142
A7	1365.3	1251.3	Tyr	d-Lys	Trp	Phe	Glu	Ser(β-d-Glc)-Ser(β-d-Glc)-CONH_2_	3.44 ± 0.9	1859 ± 423	10000	540	2904
A8	954	839.4	Tyr	d-Lys	Trp	Phe	Glu	Ser-CONH_2_	0.77 ± 0.4	341 ± 71	1263 ± 145	441	1633

a Seven glycosylated endomorphin analogs were assessed for binding to mu, delta, and kappa (MOR (μ), DOR (δ), and KOR (κ)) opioid receptors. Two non-glycosylated controls were included in the assays: ZH853 (ZH) and A8 with the C-terminal Gly replaced with a Ser, to which the glycosides were coupled in the test compounds. *K*_i_’s and selectivity are shown. Values are mean ± SEM of 3 replicate assays.

Based on receptor binding results and pilot antinociception studies where responses were below those of other glycopeptides and reference compounds, A3–A6 were not studied further at this time. A1, A2, and A7 were further characterized.

Opioid-receptor-specific functional activity was assessed in cyclicAMP (cAMP) assays by PDSP. Table 3 shows that ZH853 and all three glycopeptides showed potent, selective, full efficacy responses at MOR. ZH853 showed greater selectivity, efficacy, and especially potency, than in GTPγS assays.^8^ All compounds showed efficacies >70% for DOR and KOR, but affinities for these receptors were generally more than 100-fold lower than for MOR.

**Table 3:** Functional Activity at Opioid Receptors^a^

	**IC50 (nM)**			**Selectivity**		Potency vs ref*			Efficacy vs ref*		
	**MOR (μ)**	**DOR (δ)**	**KOR (κ)**	**δ/μ**	**κ/μ**	**MOR**	**DOR**	**KOR**	**MOR**	**DOR**	**KOR**
ref*	2.68 ± 0.82	0.39 ± 0.06	0.07 ± 0.02								
ZH853	0.07 ± 0.04	377 ± 166	2274 ± 273	5220	31460	37.1	0.0010	0.00003	1.41	0.92	0.73
A1	1.71 ± 0.58	168 ± 65	97 ± 27	98	57	1.6	0.0023	0.00069	1.04	0.84	0.91
A2	0.81 ± 0.26	990 ± 240	368 ± 26	1226	456	3.3	0.0004	0.00018	1.20	0.73	0.86
A7	1.12 ± 0.41	3730 ± 1516	426 ± 32	3317	378	2.4	0.0001	0.00016	1.09	1.07	0.84

a Gi signaling (inhibition of cAMP) was tested in opioid-receptor-expressing HEK cells by PDSP. ZH853, A1, A2, and A3 were compared to reference compounds (ref*) DAMGO, DADLE, and Salvinorin for MOR, DOR, and KOR activation, respectively. Values are means and SEMs of 3–6 assays.

## Tail Flick (TF)

**Figure 2:**
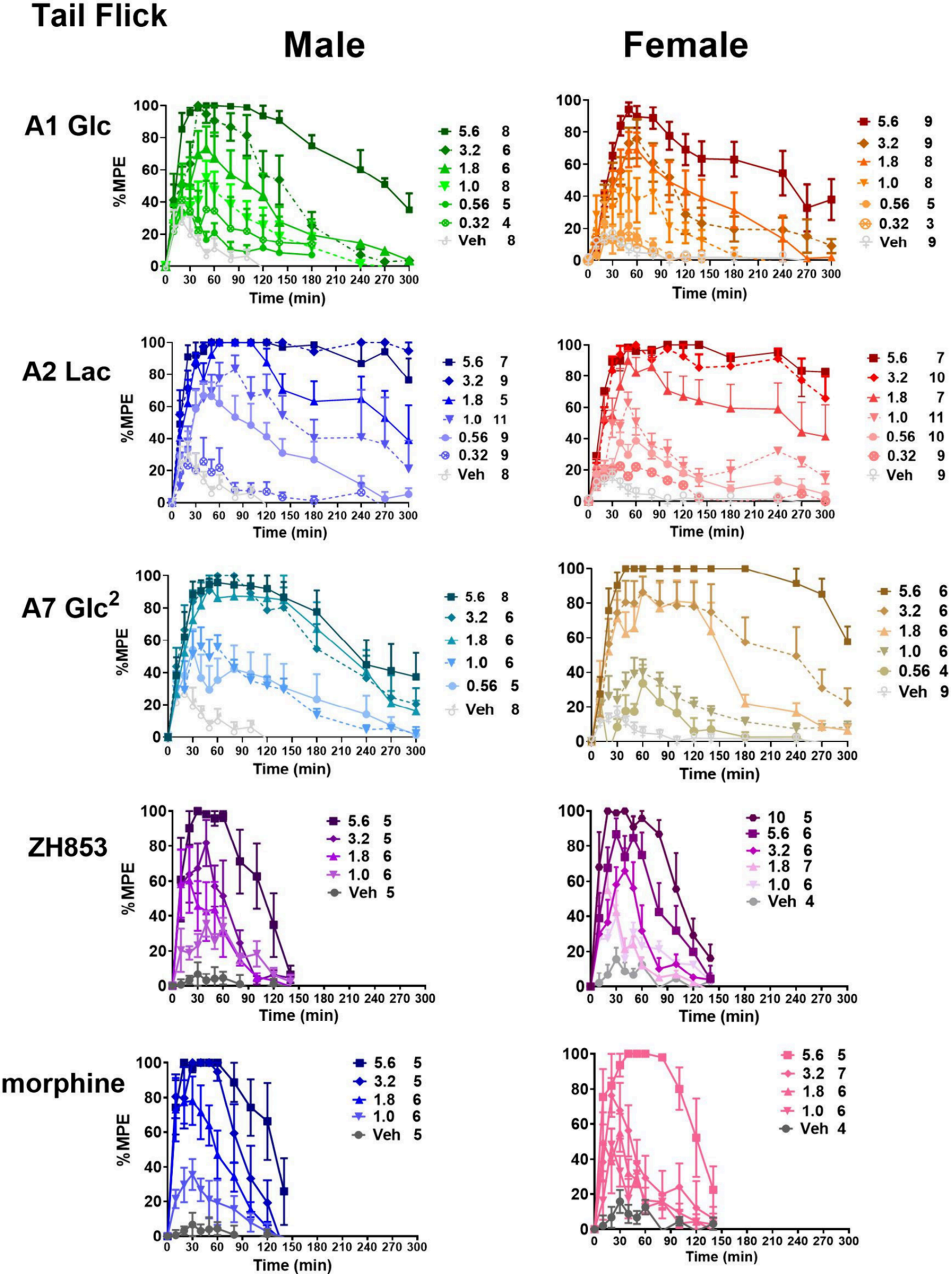
Time course of tail flick (TF) antinociception. Three glycosylated EM analogs were selected for TF testing in male and female CD-1 mice and compared to two reference compounds, ZH853 and morphine. All compounds produced dose-dependent antinociception, expressed as % maximum possible effect (%MPE). The legend shows the doses used (mg/kg s.c.), followed by the total number of animals for the time course and dose–response curves. While values for all doses of the reference compounds returned to baseline within 3 h, responses to the higher doses of glycopeptides, particularly lactoside A2, remained high at the 5 h limit of the test. The number of animals in each group is shown to the right of the doses.

**Figure 3:**
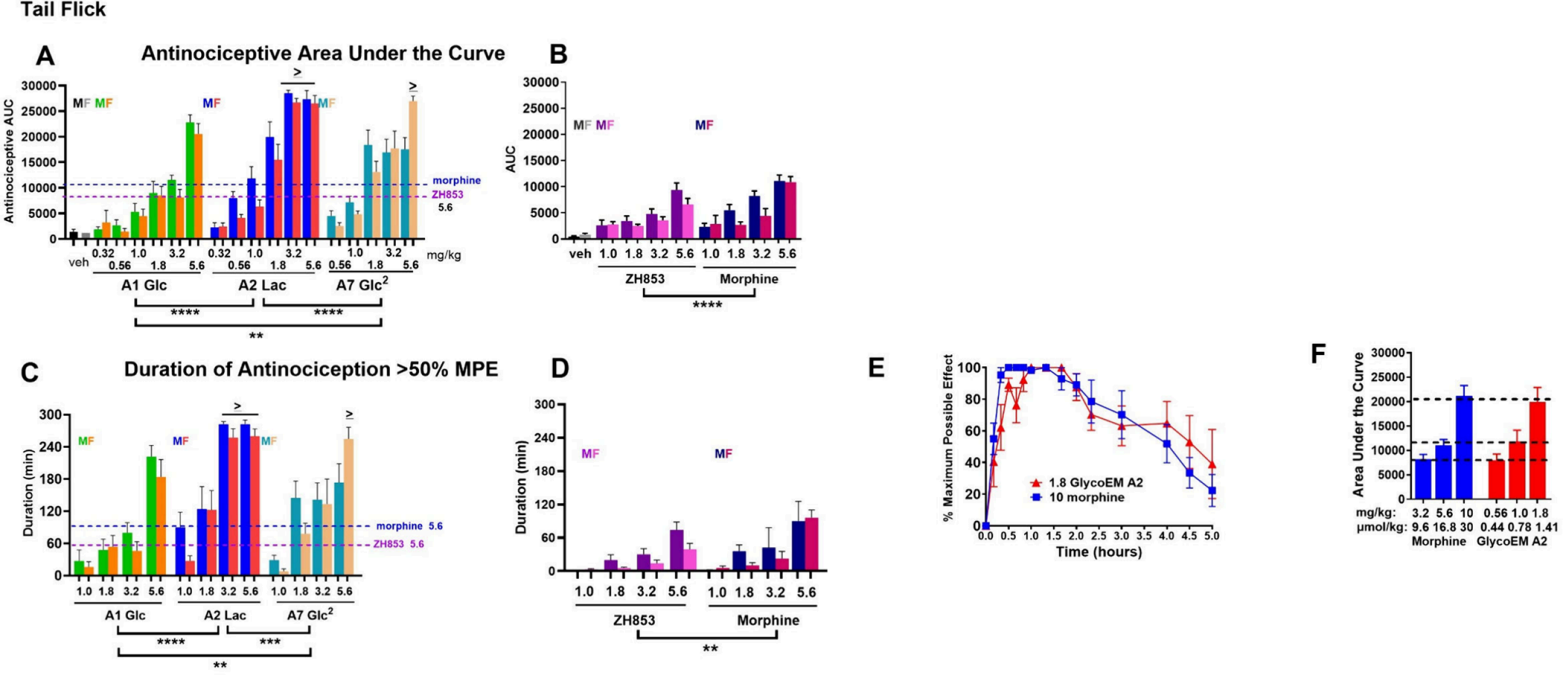
Area under the curve (AUC) and duration of TF antinociception. Significant dose-dependent increases in the antinociceptive AUC were observed for the glycopeptides (A) and reference compounds (B; *X*-axis scaled as in A for reference). Significant main effects of drug showed that lactoside A2 produced greater AUC values than all other compounds, that all three glycopeptides showed greater AUC than the reference compounds (*p* < 0.0001), and that significant differences were observed among all compounds with an overall rank order of effectiveness as A2 > A7 > A1 > morphine > ZH853. In general, female mice scored lower than males. For morphine, this resulted in a main effect of sex. However, a dose×sex interaction was observed for the double glucoside A7, where females scored better than males at the high dose but worse at low doses. AUC values for the mean male and female responses to the top dose (5.6 mg/kg) of controls ZH853 and morphine are shown as dashed lines in panels A and C for reference and indicate a minimum of 2.5- (>25000 vs 10000 AUC) to 3-fold (1.8 mg/kg A2 > 5.6 mg/kg) greater effectiveness of A2 over reference compounds. These differences are conservative as indicated by “>” above bars where responses >50% MPE were maintained at the 5 h maximum test time for the top 2 doses of A2. Duration of antinociception >50% MPE (C, D) showed similar statistical results but provided a direct index of duration and reflects a minimum 3- to 5-fold greater duration of antinociception relative to controls at the same dose. Panel E shows that a separate group of male mice (*n* = 6) tested at 10 mg/kg morphine (MS) produced peak effect, time course, duration, and AUC responses (total antinociception) well matched to 1.8 mg/kg A2, indicating that A2 provides antinociception equal to morphine at a dose 5.55-fold lower. Panel F: AUC for matched doses of morphine and A2 expressed as mg/kg and umol/kg. The latter indicates equi-antinociceptive doses for A2 are 21.8-fold lower than morphine on a molar basis. “>” = responses >50% MPE maintained at the 5 h maximum test time. (****, **p < 0.0001, *p* < 0.01). n’s as in Figure 2 except for 3.2 and 5.6 mg A2 and 5.6 mg of A1 (*n* = 4–5) as described in the Methods section in the Supporting Information.

Three glycosylated EM analogs (A1, A2 and A7) were selected for TF testing in male and female CD1 mice and compared to two reference compounds, ZH853 and morphine. Figure 2 shows the time-course of antinociception in the tail flick test and Figure 3 shows the area under the curve (AUC) and duration (%MPE > 50%) of antinociception. Three-way analyses (drug×dose×sex) of the AUC for each pair of compounds showed the expected significant effects of dose (*p* < 0.0001) for all drugs, reflecting significant dose dependence. Significant effects of drug were also observed (*p* < 0.0001), reflecting the overall rank order of effectiveness as A2 > A7 > A1 > morphine > ZH853. A main effect of sex (*p* < 0.05) was observed in the 3-way A1 vs A2 comparison, reflecting the lower overall values for females for these two compounds. The A1 vs A7 comparison revealed a dose×drug×sex interaction (*p* < 0.05) reflecting the sharper increase in female scores for A7, including scores greater than males at the top dose. However, two-way analyses of dose×sex for each compound showed no significant main effect of sex for the glycopeptides.

The double glucoside A7 showed a dimorphic dose×sex effect (*p* < 0.01) in the tail flick test, confirming the steep rise in responsiveness by females described above. A significant effect of sex (*p* < 0.05) was seen in the 3-way comparison of morphine and ZH853, with subsequent 2-way drug×dose analyses showing a significant effect (*p* < 0.05) and a statistical tendency (*p* < 0.1) of sex differences for morphine and ZH853 respectively. As expected, similar statistical effects were observed for duration >50% MPE.

In summary, overall significant differences among the compounds showed a rank order of effectiveness as A2 (Lac) > A7 (Glc2) > A1 (Glc1) > morphine > ZH853. The effectiveness and duration of antinociception for A2 males and females, and A7 females, is a conservative estimate since several animals in each group had %MPE scores >50% at the maximum test time at 5 h. To determine relative effectiveness more accurately, a separate group of mice (*n* = 6) was tested with 10 mg/kg morphine and compared to 1.8 mg/kg A2, doses producing full (100%MPE) antinociception and a duration >3 h. Figure 3E and F shows that these doses, as well as 2 matched lower doses, produce well-matched time course and AUC data and indicate that A2 produces total antinociceptive effects equal to morphine at a dose 5.55-fold lower on a mg/kg basis and 21.8-fold lower on a molar basis.

Effects of sex reflected generally lower scores for females. This occurred across doses for A1 and ZH853, while for A2, A7, and morphine, female scores were lower at low doses but closer to males at high doses. For A7, female scores showed a steep increase and exceeded those of males at 5.6 mg/kg, reflected by a significant drug×dose×sex interaction in the comparison of A1 and A7.

**Figure 4:**
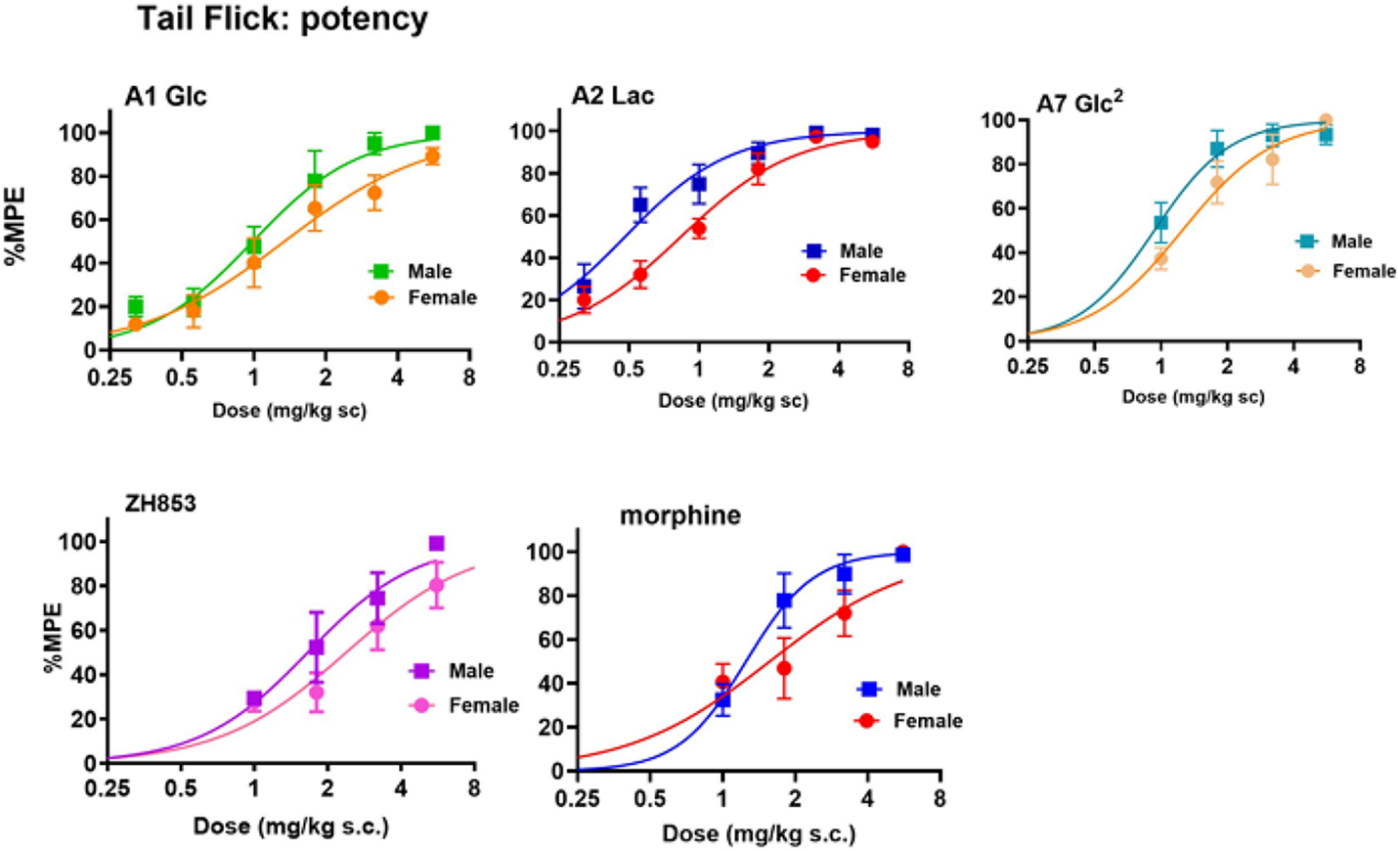
TF dose–response curves. Percent MPE values at peak response times as described in methods were used to determine the ED_50_. Male and female (M, F) dose–response curves for each compound are shown.

Dose–response curves are shown in Figure 4 and potencies (ED_50_) in Table 4. The rank order of potency is the same as that of the AUC and duration described above (A2 > A7 > A1 > morphine > ZH853) for mg/kg calculations, but on a molar basis (μmol/kg), the rank order of morphine and ZH853 are reversed. A2 is 2- to 3-fold more potent than controls for mg/kg and 4- to 8-fold for μmol/kg. The potency was lower for females than males for all compounds, with significant effects for A2 and ZH853. None of the ratios, however, exceeded 2-fold. For both males and females, A2 was significantly more potent (*p* < 0.01–0.0001) than all other compounds except for male A2 vs A7 showing a statistical tendency (*p* = 0.0517). Lactoside A2 potency relative to controls was 2–3-fold for mg/kg and 4–8-fold on a molar basis (μmol/kg).

**Table 4:** Tail Flick Potencies (ED_50_) in mg/kg (Top) and μmol/kg (Bottom), Listed in Rank Order of Potency^a^

	**ED**_**50**_ (mg/kg)					**Potency vs control**	
	**Male**	**Female**	**Mean** M&F	**Mean** F/M	**MvF signif**	**ZH853/ analog**	**MS/ analog**
A2	**0.48** ± 0.05	**0.84** ± 0.06	0.66	1.74	0.0001	3.06	2.14
A7	**0.93** ± 0.14	**1.25** ± 0.16	1.09	1.34	0.076	1.85	1.29
A1	**0.99** ± 0.01	**1.34** ± 0.19	1.17	1.35	0.073	1.73	1.21
morphine	**1.25** ± 0.11	**1.57** ± 0.35	1.41	1.25	0.266	1.43	
ZH853	**1.66** ± 0.23	**2.38** ± 0.30	2.02	1.44	0.043		0.70


	**ED**_**50**_ (μmol/kg)					**Potency vs control**	
	**Male**	**Female**	**Mean** M&F	**Mean** F/M	**MvF signif**	**ZH853/ analog**	**MS/ analog**
A2	**0.38** ± 0.04	**0.66** ± 0.05	0.52	1.74	0.0001	4.48	8.12
A7	**0.68** ± 0.11	**0.91** ± 0.12	0.80	1.34	0.076	2.91	5.28
A1	**0.89** ± 0.09	**1.20** ± 0.17	1.08	1.43	0.073	2.16	3.91
ZH853	**1.91** ± 0.27	**2.74** ± 0.36	2.33	1.44	0.266		1.81
morphine	**3.74** ± 0.39	**4.69** ± 1.08	4.22	1.25	0.043	0.55	

a Relative potencies for male vs female, and for each glycopeptide vs both control compounds are shown. Values are mean ± SEM. *n*’s are shown in Figure 2.

**Figure 5:**
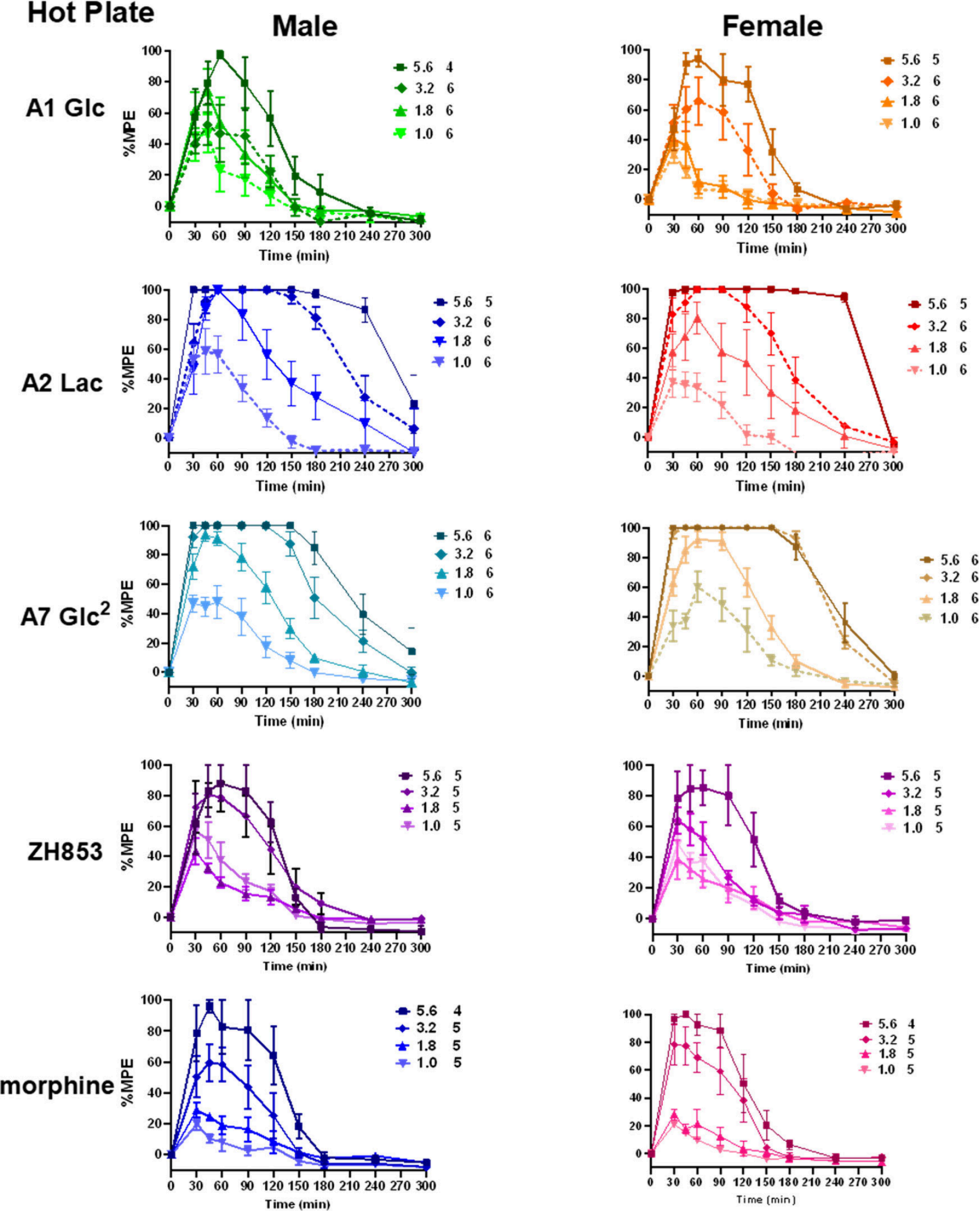
Time course of hot plate (HP) antinociception. The three analogs and two reference compounds tested in the tail flick test were also tested for HP responses in male and female DBA mice for 5 h. All compounds produced dose-dependent antinociception, expressed as %maximum possible effect (%MPE). Doses (mg/kg) and +n’s are shown in the legends.

**Figure 6:**
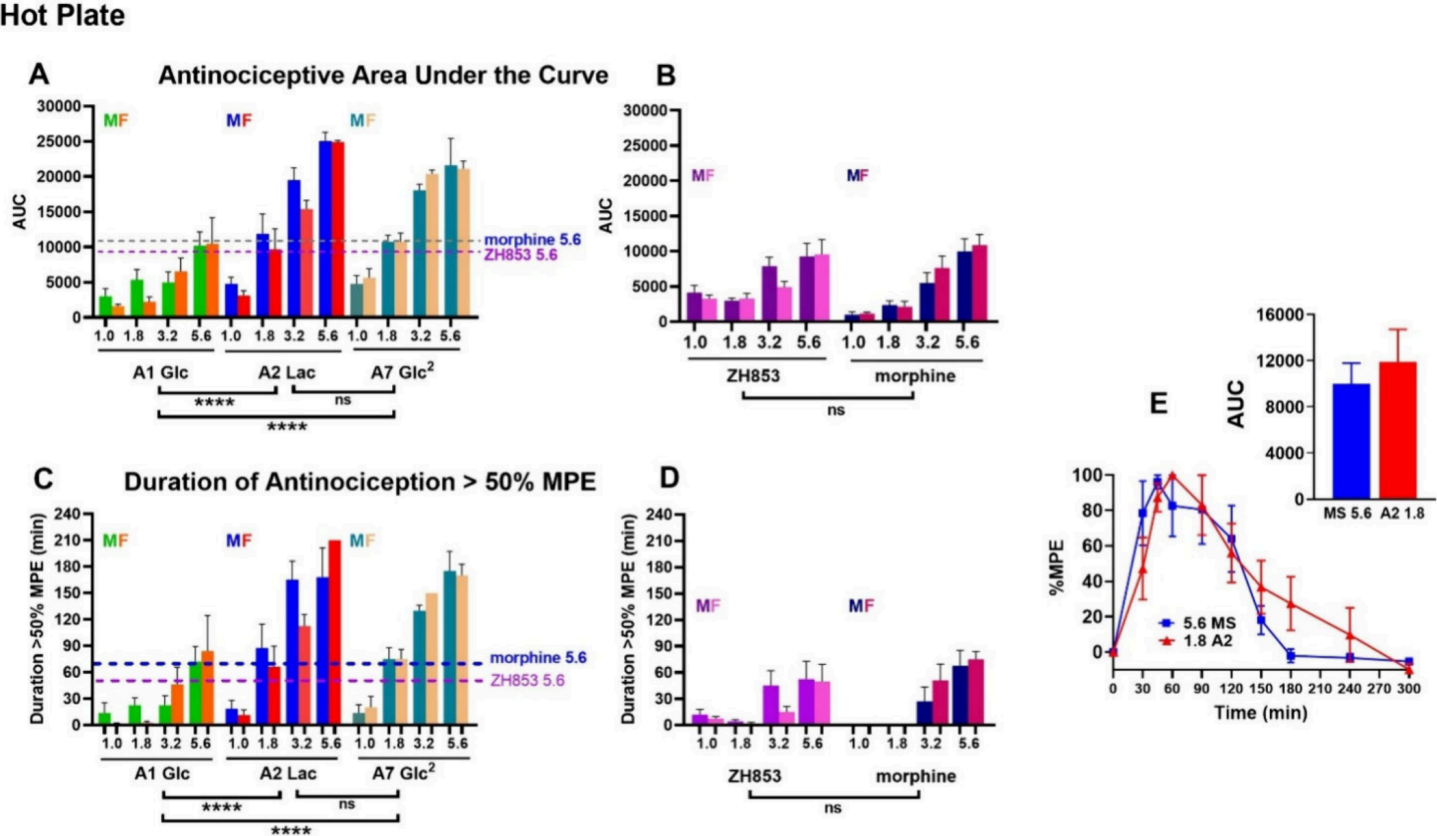
Area under the curve (AUC) and duration of HP antinociception. As with the TF test, all compounds produced dose-dependent increases in duration of antinociception, as shown for AUC (A, B) and duration >50% MPE figures (C, D). Main effects of drug were not significant between A2 and A7, or among A1 and the reference compounds ZH853 and morphine, indicating two tiers of effectiveness, with A2 and A7 producing higher overall antinociceptive effects than the other compounds. Sex differences (and interactions) were not significant, indicating similar effects for both sexes for all compounds in this test. Values for the mean male and female responses to the top dose (5.6 mg/kg) of ZH853 and morphine are shown as dashed lines in panels A and C and indicate ∼3-fold greater effectiveness of A2 (1.8 vs 5.6 mg/kg for controls). Panel E directly illustrates that 1.8 mg/kg A2 provides a similar peak effect, duration, and AUC to 5.6 mg/kg morphine, confirming a 3.1-fold greater effectiveness. (**** *p* < 0.0001, *n*’s as in Figure 5).

Figure 5 shows the time course of antinociception in the hot plate (HP) and Figure 6 shows the AUC (A, B) and duration (C, D). As with the tail flick test, analogs A2 and A7 produced robust antinociception in this test, consistent with the concept that these compounds activate central mechanisms regulating pain. In contrast to the TF test, however, the responses of animals given A1 were not significantly greater than those of reference compounds ZH853 and morphine, and no significant sex differences were observed. Three-way analyses (drug×dose×sex) of hot plate (HP) area under the curve (AUC) data for each pair of compounds confirmed dose-dependence with a significant effect of dose (*p* < 0.0001 in all cases). The effect of drug was not significant between A2 vs A7 or between A1, ZH853, and morphine, indicating two tiers of effectiveness, with A2 and A7 producing greater antinociceptive effects than the other 3 compounds. A significant effect of dose×drug for A2 or A7 compared to the other compounds indicated a greater rate of increase in response to increased dose. Sex differences (and interactions) were not significant, indicating similar effects for both sexes for all compounds on this measure. One-way analyses at each dose across groups showed that A2 and A7 produced a significantly greater AUC than A1, morphine and ZH853 at 3 of the four doses tested: 1.0,1.8, 3.2, and 5.6 mg/kg.

As shown in Figure 6, the AUC ranges for 1.8 mg/kg of A2 and A7 were similar to those of 5.6 mg/kg of A1, ZH853, and morphine, indicating ∼3-fold greater effectiveness of A2 and A7. Figure 6E shows this comparison directly as 1.8 mg/kg of A2 and 5.6 mg/kg morphine produce similar peak effects, time course, and area under the curve, supporting the estimate of 3-fold greater effectiveness of A2. Duration >50% MPE (C, D) showed similar statistical effects.

**Figure 7:**
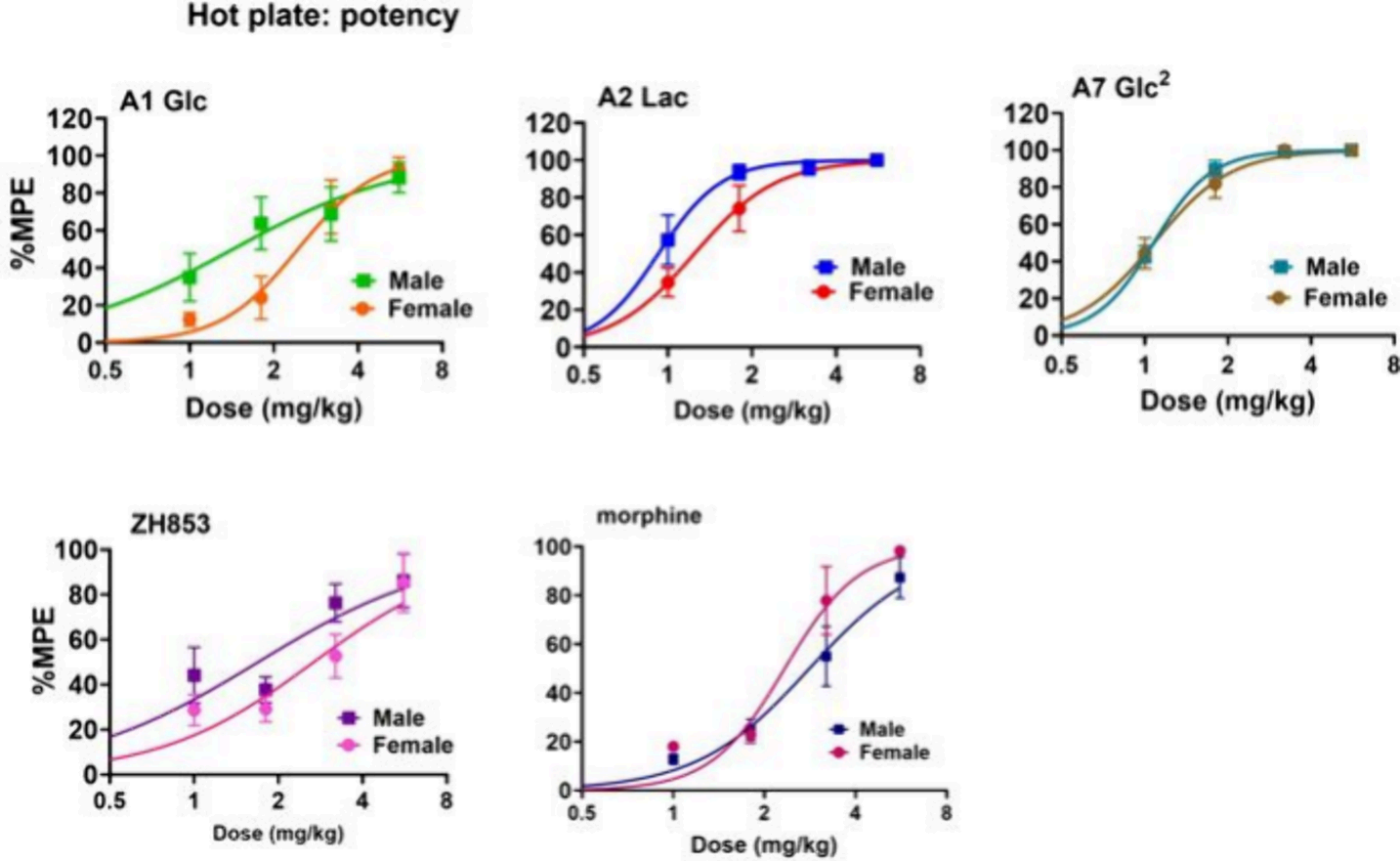
Hot plate (HP) dose–response curves. Percent MPE values at peak response times as described in methods were used to determine the ED_50_ values. Male and female (M,F) dose–response curves for each compound are shown.

Dose–response curves are shown in Figure 7 and relative potency (ED_50_) of the compounds in Table 5. The rank order of potency is the same as that of the total antinociception (AUC, duration) described above for TF except that ZH853 was more potent than morphine for both mg/kg and μmol/kg values (A2 > A7 > A1 > ZH853 > morphine). The potency was lower for females than males for all compounds, with significant effects for A1, A2 and ZH853. None of the ratios, however, exceeded 2-fold. Males and females given A2 and A7, but not A1, showed significantly greater potency than the ZH853 and morphine controls.

**Table 5:** Potency of Analogs in the Hot Plate Assay^a^

	**ED**_**50**_ mg/kg					**Potency vs control**	
	**Male**	**Female**	**Mean** M&F	**Mean** F/M	**MvF signif**	**ZH853/ analog**	**MS/ analog**
A2	**0.92** ± 0.10	**1.24** ± 0.10	1.08	1.35	0.007	2.02	2.38
A7	**1.08** ± 0.04	**1.08** ± 0.08	1.08	1.00	0.950	2.02	2.38
A1	**1.43** ± 0.34	**2.44** ± 0.22	1.93	1.71	0.008	1.13	1.33
ZH853	**1.69** ± 0.34	**2.67** ±. 039	2.18	1.57	0.030		1.18
morphine	**2.82** ± 0.24	**2.33** ± 0.18	2.57	0.83	0.105	0.85	


	**ED**_**50**_ μmol/kg					**Potency vs control**	
	**Male**	**Female**	**Mean** M&F	**Mean** F/M	**MvF signif**	**ZH853/ analog**	**MS/ analog**
A2	**0.72** ± 0.08	**0.97** ± 0.09	0.85	1.35		2.95	9.07
A7	**0.79** ± 0.03	**0.79** ± 0.06	0.79	1.00		3.18	9.76
A1	**1.28** ± 0.34	**2.18** ± 0.22	1.73	1.71		1.45	4.46
ZH853	**1.94** ± 0.39	**3.07** ± 0.44	2.51	1.58			3.07
morphine	**8.43** ± 0.73	**6.99** ± 0.52	7.71	0.83		0.33	

a ED_50_ values calculated from doses expressed both as mg/kg (top) and μmol/kg (bottom) are shown. Compounds are listed by rank order of potency and show that A2 and A7 are ∼2-fold more potent than controls for mg/kg and 3- to 9-fold for μmol/kg values.

In summary, glycosylated endomorphin analogs were synthesized and tested for opioid receptor binding and antinociception in male and female mice in tail flick and hot plate tests. A nonglycosylated reference compound, ZH853, showed the highest MOR affinity (subnanomolar K_*i*_) followed by a nonglycosylated Ser analog (A8). Glycosylation reduced the affinity for MOR ∼5-fold for analogs A1, A2, A6 and A7. For A2, the specificity was slightly decreased relative to DOR, but slightly increased relative to KOR. By contrast, A7 showed dramatically reduced DOR and KOR, resulting in a highly selective MOR agonist. ZH853, A1, A2 and A7 were potent, fully efficacious MOR-selective agonists in cAMP assays. Disaccharide A2 and double glucoside A7 provided greater receptor selectivity, potency, and total antinociception, than a single carbohydrate moiety (A1).

Despite some loss of MOR binding affinity, the antinociceptive effectiveness increased for A2 and A7 relative to reference compounds. Doses (5.6 mg/kg) at which morphine produced ∼2 h of antinociception in the tail flick test provided over 5 h of pain relief by one of the analogs (A2). Comparable antinociception occurred at A2 doses 5-fold lower (20-fold on a molar basis) than morphine doses. As shown for several peptides described in the introduction and for A1 in this study, glycosylation increases penetration of the BBB. This likely contributed to the increased antinociception.

In general, A2 and A7 were highly effective in both males and females. There were some significant differences in the effect of sex in 3-way analyses (drug×dose×sex) in the tail flick test and lower (<2×) potencies in tail flick and hot plate tests. These reflect generally lower scores for females. This is consistent with studies showing that in rodents, opioids tend to show lower analgesic potency in females than in males.^24−26^ However, 2-way analyses (dose×sex) for each compound showed no significant effect of sex for the glycopeptides, indicating that the analogs could serve as effective pain medications for both males and females

Earlier studies with glycosylated EMs have produced some centrally mediated antinociception,^17,22^ but the combination of cyclization to produce stability in conjunction with glycosylation to produce enhanced bioavailability has produced highly promising drug candidates. The results provide additional evidence for the utility of glycopeptide drugs^13^ and strongly support further study of the glycosylated EMs for clinical application.

## Supporting Information Available

Synthesis, chemical characterization, and modeling of analogs, *in vivo* microdialysis, and antinociception methods (PDF)

## Abbreviations

A1–A8: Analogs 1–8
AUC: Area under the curve
BBB: Blood–brain barrier
cAMP: Cyclic adenosine monophosphate
CPP: Conditioned place preference
DOR: Delta opioid receptor
EM(s): Endomorphin(s)
HP: Hot plate
KOR: Kappa opioid receptor
MOR: Mu opioid receptor
%MPE: %Maximum possible effect
SA: Self-administration
TF: Tail flick
