# Supplementary material for: Cyclic Glycopeptide Analogs of Endomorphin-1 Provide Highly Effective Antinociception in Male and Female Mice
Source: ACS Med Chem Lett. 2024 Sep 17;15(10):1731–40. doi: 10.1021/acsmedchemlett.4c00315 (PMC11472388; doi:10.1021/acsmedchemlett.4c00315)
Supplement: Supplementary file 1 — ml4c00315_si_001.pdf [file ml4c00315_si_001.pdf]

Cyclic Glycopeptide Analogs of Endomorphin-1 Provide Highly Effective Antinociception in Male and Female Mice

*James E. Zadina[1,2,3,4], Lajos Z. Szabo[5], Fahad Al-Obeidi[5], Xing Zhang[1,4], Leticia Ferreira Nakatani[1,4], Chidiebere Ogbu[5], M. Leandro Heien[5], Torsten Falk[6], Mitchell J. Bartlett[7] and Robin Polt[5]

[1]Departments of Medicine & [2]Pharmacology, and [3]Brain Institute, Tulane University School of Medicine, New Orleans, LA 70112 *jzadina@tulane.edu

[4]SE Louisiana Veterans Health Care System, New Orleans, LA 70119

[5]Department of Chemistry & Biochemistry, The University of Arizona, Tucson, AZ 85721,
[6]Department of Neurology, The University of Arizona, Tucson, AZ

[7]Departments of Surgery and Neurosurgery, The University of Arizona, Tucson, AZ 85724

*Corresponding Author

Supporting Information on Methods

METHOD **Page Number**

Synthesis of endomorphin analogs by solid phase peptide synthesis……..........……. S2

Figs S1a—g. HPLC and mass spectrometry profiles for the glycoside analogs........S4–S10

Fig S2. *In silico* model…………………………………………………...............................S11

Animals and *in vivo* methods (Tail flick and Hot Plate antinociception) ……….........S12–17

Fig S3. Microdialysis and Pharmacokinetics Experiment Scheme…....................…....…S17

Fig S4. Time Course of *In Vivo* Blood Brain Barrier Penetration and Plasma Pharmacokinetics……………………………………………………………………………….S18

Fig S5. Area Under the Curve (AUC) of *In Vivo* Blood Brain Barrier Penetration and Plasma Pharmacokinetics…………………………………………………………………….S19

References………………………………………………………………………………….….S15

Peptide Synthesis: The unglycosylated cyclic peptide ZH853 was custom synthesized as the acetate salt and certified as >95% purity by Anaspec (Fremont, CA). Glycopeptides **A1**–**A7** and the unglycosylated control peptide **A8** were synthesized and estimated >95% purity by HPLC.

Synthesis of the Endomorphin Analogs. The glycosylated cyclic endomorphin analogues (6 or 7 AA residue peptides) were synthesized using standard Fmoc procedures for solid phase synthesis (SPPS) with our Prelude® peptide synthesizer. The amino acid glucoside (Fmoc-Ser[O-β-D-Glc$(OAc)_4$]-OH, and the lactoside Fmoc-Ser[O-β-Lact$(OAc)_7$]-OH) were both prepared using published procedures.[1] The 1st residues on the resin, either Fmoc-Gly-OH, Fmoc-Ser[O-β-D-Glc$(OAc)_4$]-OH, or Fmoc-Ser[O-β-Lact$(OAc)_7$]-OH were manually coupled to MBHA Rink Amide resin with Cl–HOBt and DIC in NMP. After the initial amino acid coupling the resin was capped with Ac_2O and DIEA in DCM to provide a final resin loading of 0.5 to 0.7 mmol/g of Fmoc-amino acid. The subsequent Fmoc-protected amino acids were automatically coupled with HBTU and NMM using the same protocol to achieve the desired sequence. Cyclization was achieved with the orthogonally protected side-chain residues Fmoc-Glu(All) and Fmoc-D-Lys(Alloc) at the cyclization site.[2] The cyclization was attempted both immediately after the Fmoc-D-Lys(Alloc) coupling, and on the completed peptide while still on the resin– the latter method was superior. The final amino acid Tyrosine was coupled as the Boc-Tyr(tBu)-OH to obviate the final Fmoc group removal. The Allyl and Alloc protecting groups were removed with $Pd(PPh_3)_4$ and $PhSiH_3$ in DCM. The most effective lactam ring closure method was on the resin in the presence of PyClock with DIEA in NMP solvent.[2] The acetyl groups were removed from the sugar moieties with hydrazine hydrate ($H_2NNH_2 \cdot H_2O$) in NMP while the glycopeptide was still on the resin. An acidic cleavage cocktail containing TFA was used to simultaneously remove the amino acid sidechain protecting groups, the N-terminal Boc group, and cleave the cyclic glycopeptide from

the resin. The resultant crude peptides were purified by reverse phase HPLC using a 55 mm preparative column. The purity of each product was verified using analytical reverse HPLC and the glycoside structures were confirmed with ESI mass spectrometry. HPLC and MS data shown below.

A1 (Glucoside) 2LS1753

Sequence: Tyr-c-[*D*-Lys-Trp-Phe-Glu]-Ser(ß-*D*-Glc)-$CONH_2$

Structure:

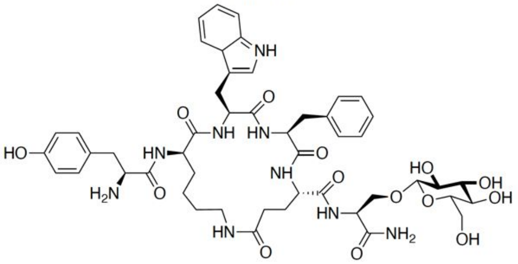

Chemical Formula: $C_{49}H_{64}N_9O_{14}$
Exact Mass: 1002.45727
Molecular Weight: 1003.1

Mw: **1117** (as TFA salt)

Solubility: H_2O, Saline

Storage Condition: Dry condition, < 5°C

HPLC:

Data File: c:\star\data\2laj\2ls186\2ls186-final-pure
Channel: 2 = 280.00 nm RESULTS
Sample ID: 2ls186-5
Operator (Inj): polt lab
Injection Date: 05/28/2021 02:43:06 PM
Injection Method: c:\star\method\lajos\lsz_2_analit.mth
Run Time (min): 23.013
Workstation:
Instrument (Inj): varian

Operator (Calc): polt lab
Calc Date: 05/28/2021 04:01:20
Times Calculated: 2
Calculation Method: c:\docume~1\poltlab\locals~1\temp\~2ls1
Instrument (Calc): varian
Run Mode: Analysis
Peak Measurement: Peak Area
Calculation Type: Percent
Calibration Level: N/A
Verification Tolerance: N/A

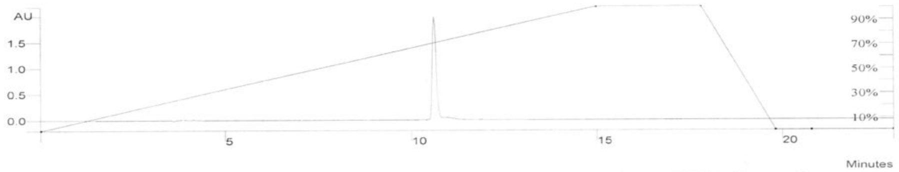

Peak No	Peak Name	Result ()	Ret. Time (min)	Time Offset (min)	Area (counts)	Rel Ret Time	Sep. Code	Width 1/2 (sec)	Status Codes	Group
1		99.6217	10.616	0.000	76131552	0.00	BB	6.9		0
2		0.3783	10.957	0.000	289105	0.00	TS	0.0		0
	Totals	100.0000		0.000	76420656					

Mass spectra:

Generic Display Report

Analysis Info
Analysis Name: D:\Data\2021_02\LZS-3918428\LZS-3918428_2ls175_37.d
Method: EmptyAcquisition.ms
Sample Name: 2ls175_37
Comment: Dissolved in 200ul 50%ACN, then 100x dil with 50%ACNH2O

Acquisition Date: 2/9/2021 4:28:46 PM
Operator: KK
Instrument: amaZon SL

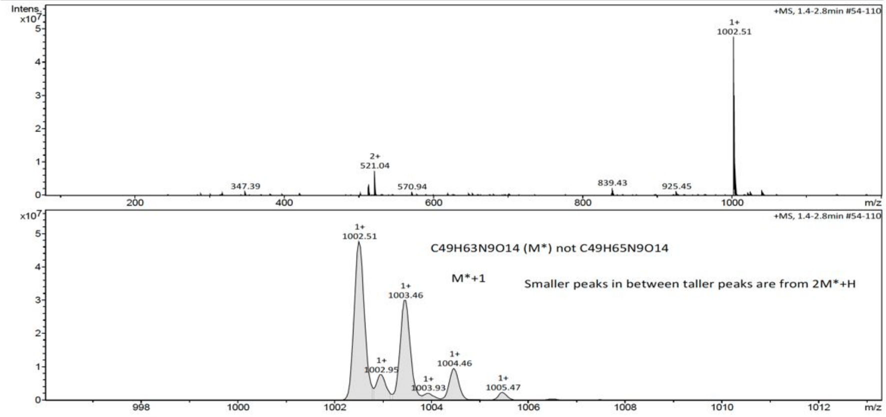

Bruker Compass DataAnalysis 4.2 printed: 2/9/2021 4:34:22 PM by: KK Page 1 of 1

Figure S1a

A2 (lactoside) 2LS1754 (2LS1774)

Sequence: Tyr-c-[*D*-Lys-Trp-Phe-Glu]-Ser(ß-Lact)-CONH$_2$

Structure:

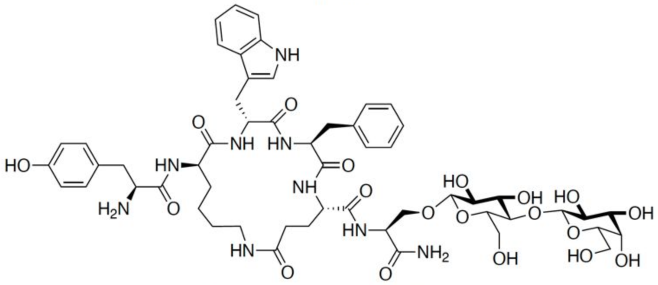

Chemical Formula: $C_{55}H_{73}N_9O_{19}$
Exact Mass: 1163.50227
Molecular Weight: 1164.23300

Mw: **1278.2** (as TFA salt)

Solubility: H_2O, Saline

Storage Condition: Dry condition, < 5°C

HPLC:

Data File:	c:\star\data\2laj\2ls186\2ls186-final-pure'	Operator (Calc):	polt lab
Channel:	2 = 280.00 nm RESULTS	Calc Date:	05/28/2021 04:06:05
Sample ID:	2ls186-6	Times Calculated:	2
Operator (Inj):	polt lab	Calculation Method:	c:\docume~1\poltlab\locals~1\temp\~2ls1
Injection Date:	05/28/2021 03:09:26 PM	Instrument (Calc):	varian
Injection Method:	c:\star\method\lajos\lsz_2_analit.mth	Run Mode:	Analysis
Run Time (min):	23.013	Peak Measurement:	Peak Area
Workstation:		Calculation Type:	Percent
Instrument (Inj):	varian	Calibration Level:	N/A
		Verification Tolerance:	N/A

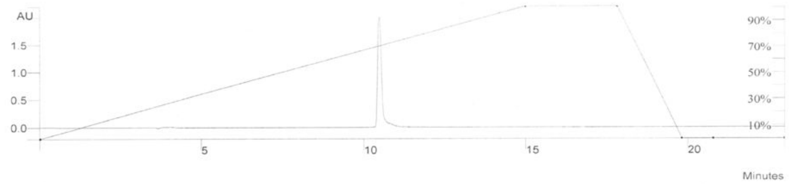

Peak No	Peak Name	Result ()	Ret. Time (min)	Time Offset (min)	Area (counts)	Rel Ret Time	Sep. Code	Width 1/2 (sec)	Status Codes	Group
1		100.0000	10.483	0.000	93806152	0.00	BB	7.5		0
	**Totals**	**100.0000**		**0.000**	**93806152**					

Mass spectra:

Generic Display Report

**Analysis Info**		Acquisition Date	2/9/2021 5:00:12 PM
Analysis Name	D:\Data\2021_02\LZS-3918428\LZS-3918428_2ls175_4.d		
Method	Tuning Mix.m	Operator	KK
Sample Name	2ls175_4	Instrument	amaZon SL
Comment	Dissolved in 200ul 50%ACN, then 100x dil with 50%ACNH2O		

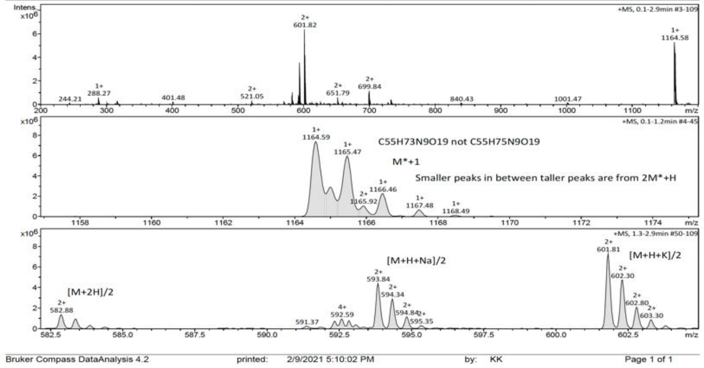

Bruker Compass DataAnalysis 4.2 printed: 2/9/2021 5:10:02 PM by: KK Page 1 of 1

Figure S1b

A3 (glucoside) 2LS175-2

Sequence: Tyr-c-[*D*-Lys-Trp-Phe-Glu]-Gly-Ser(ß-Glc)-$CONH_2$

Structure:

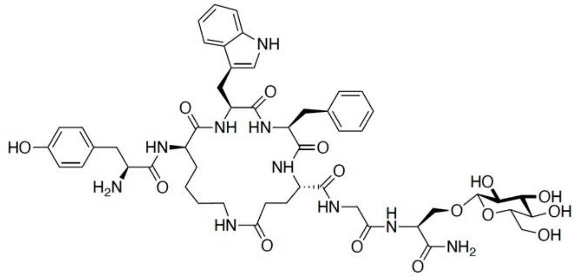

Chemical Formula: $C_{51}H_{66}N_{10}O_{15}$
Exact Mass: 1058.47091
Molecular Weight: 1059.1

Mw: **1173.1** (as TFA salt)

Solubility: H_2O, Saline

Storage Condition: Dry condition, < 5°C

HPLC:

Data File:	c:\star\data\2laj\2ls186\2ls186-final-pure	Operator (Calc):	polt lab
Channel:	2 = 280.00 nm RESULTS	Calc Date:	05/28/2021 05:33:46
Sample ID:	2ls175-2-8mg	Times Calculated:	2
Operator (Inj):	polt lab	Calculation Method:	c:\docume~1\poltlab\locals~1\temp\~2ls1
Injection Date:	05/28/2021 03:35:47 PM	Instrument (Calc):	varian
Injection Method:	c:\star\method\lajos\lsz_2_analit.mth	Run Mode:	Analysis
Run Time (min):	23.013	Peak Measurement:	Peak Area
Workstation:		Calculation Type:	Percent
Instrument (Inj):	varian	Calibration Level:	N/A
		Verification Tolerance:	N/A

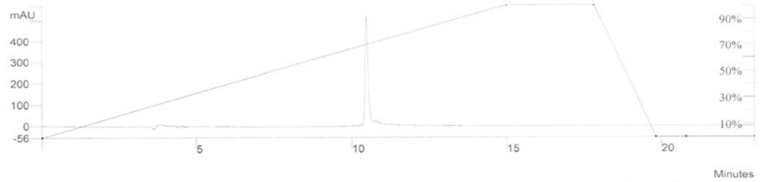

Peak No	Peak Name	Result ()	Ret. Time (min)	Time Offset (min)	Area (counts)	Rel Ret Time	Sep. Code	Width 1/2 (sec)	Status Codes	Group
1		98.6878	10.480	0.000	19592896	0.00	BB	6.4		0
2		1.3122	10.771	0.000	260516	0.00	TS	0.0		0
	Totals	100.0000		0.000	19853412					

Mass spectra:

Generic Display Report

**Analysis Info**		Acquisition Date	2/9/2021 4:08:53 PM
Analysis Name	D:\Data\2021_02\LZS-3918428\LZS-3918428_2ls175_2.d		
Method	Tuning Mix.m	Operator	KK
Sample Name	2ls175_2	Instrument	amaZon SL
Comment	Dissolved in 200ul 50%ACN, then 100x dil with 50%ACN		

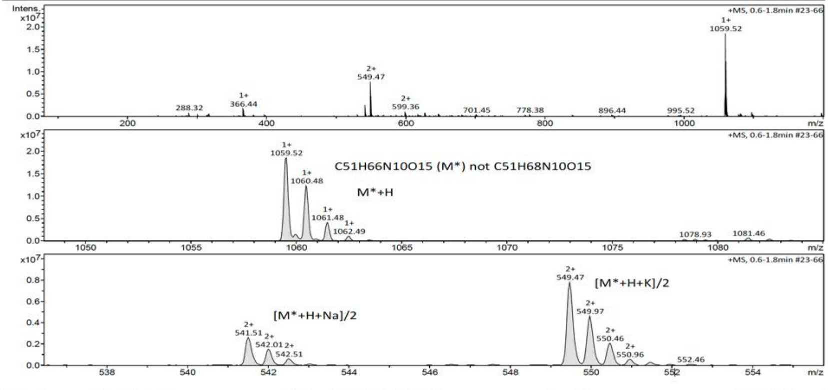

Bruker Compass DataAnalysis 4.2 printed: 2/9/2021 4:14:37 PM by: KK Page 1 of 1

Figure S1c

A4 (glucoside) 2LS186-1

Sequence: Tyr-c-[*D*-Lys-1-Nal-Phe-Glu]-Ser(ß-Glc)-CONH₂

Structure:

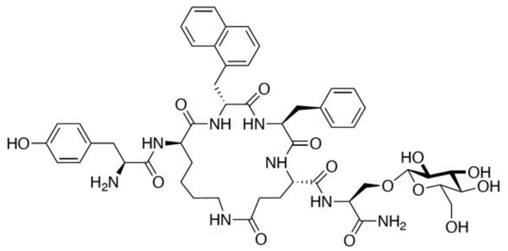

Chemical Formula: $C_{51}H_{64}N_8O_{14}$
Exact Mass: 1012.45420
Molecular Weight: 1013.1

Mw: **1127.1** (as TFA salt)

Solubility: H_2O, Saline

Storage Condition: Dry condition, < 5°C

HPLC:

Data File:	c:\star\data\2laj\2ls186\2ls186-final-pure	Operator (Calc):	polt lab
Channel:	2 = 280.00 nm RESULTS	Calc Date:	05/28/2021 01:36:01
Sample ID:	2ls186-1	Times Calculated:	2
Operator (Inj):	polt lab	Calculation Method:	c:\docume~1\poltlab\locals~1\temp\~2ls1
Injection Date:	05/28/2021 12:57:43 PM	Instrument (Calc):	varian
Injection Method:	c:\star\method\lajos\lsz_2_analit.mth	Run Mode:	Analysis
Run Time (min):	23.013	Peak Measurement:	Peak Area
Workstation:		Calculation Type:	Percent
Instrument (Inj):	varian	Calibration Level:	N/A
		Verification Tolerance:	N/A

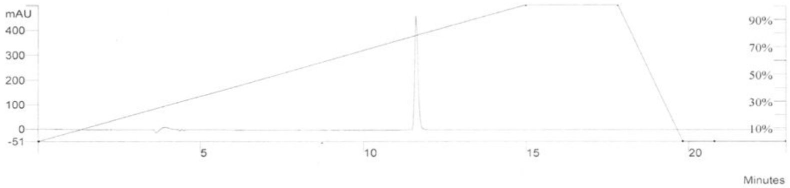

Peak No	Peak Name	Result ()	Ret. Time (min)	Time Offset (min)	Area (counts)	Rel Ret Time	Sep. Code	Width 1/2 (sec)	Status Codes	Group
1		100.0000	11.629	0.000	17093624	0.00	BB	6.6		0
	Totals	100.0000		0.000	17093624					

Mass spectra:

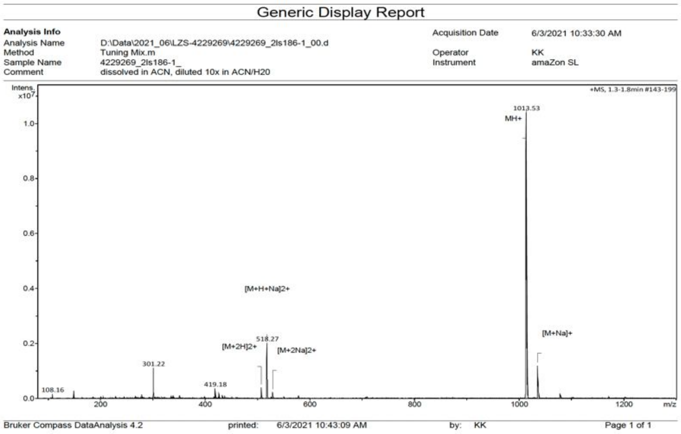

Figure S1d

A5 (lactoside) 2LS186-2

Sequence: Tyr-c-[*D*-Lys-1-*D*-Nal-Phe-Glu]-Ser(ß-Lact)-$CONH_2$

Structure:

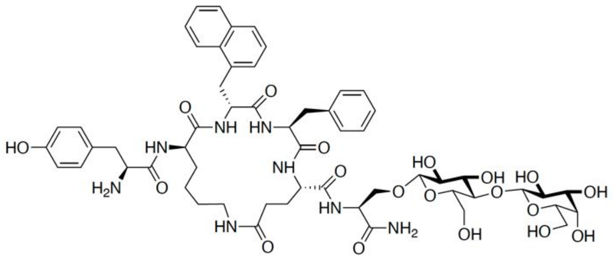

Chemical Formula: $C_{57}H_{74}N_8O_{19}$
Exact Mass: 1174.50702
Molecular Weight: 1175.3

Mw: **1289.3** (as TFA salt)

Solubility: H_2O, Saline

Storage Condition: Dry condition, < 5°C

HPLC:

Data File:	c:\star\data\2laj\2ls186\2ls186-final-pure'	Operator (Calc):	polt lab
Channel:	2 = 280.00 nm RESULTS	Calc Date:	05/28/2021 02:00:24
Sample ID:	2ls186-2	Times Calculated:	2
Operator (Inj):	polt lab	Calculation Method:	c:\docume~1\poltlab\locals~1\temp\~2ls1
Injection Date:	05/28/2021 01:24:04 PM	Instrument (Calc):	varian
Injection Method:	c:\star\method\lajos\lsz_2_analit.mth	Run Mode:	Analysis
Run Time (min):	23.013	Peak Measurement:	Peak Area
Workstation:		Calculation Type:	Percent
Instrument (Inj):	varian	Calibration Level:	N/A
		Verification Tolerance:	N/A

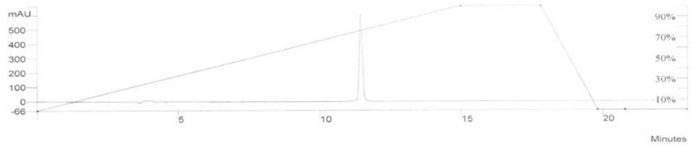

Peak No	Peak Name	Result ()	Ret. Time (min)	Time Offset (min)	Area (counts)	Rel Ret Time	Sep. Code	Width 1/2 (sec)	Status Codes	Group
1		100.0000	11.453	0.000	24482546	0.00	BB	7.0		0
	Totals	100.0000		0.000	24482546					

Mass spectra:

Generic Display Report

**Analysis Info**		Acquisition Date	6/3/2021 10:50:14 AM
Analysis Name	D:\Data\2021_06\LZS-4229269\4229269_2ls186-2_00.d		
Method	Tuning Mix.m	Operator	KK
Sample Name	4229269_2ls186-2_	Instrument	amaZon SL
Comment	dissolved in ACN, diluted 10x in ACN/H20		

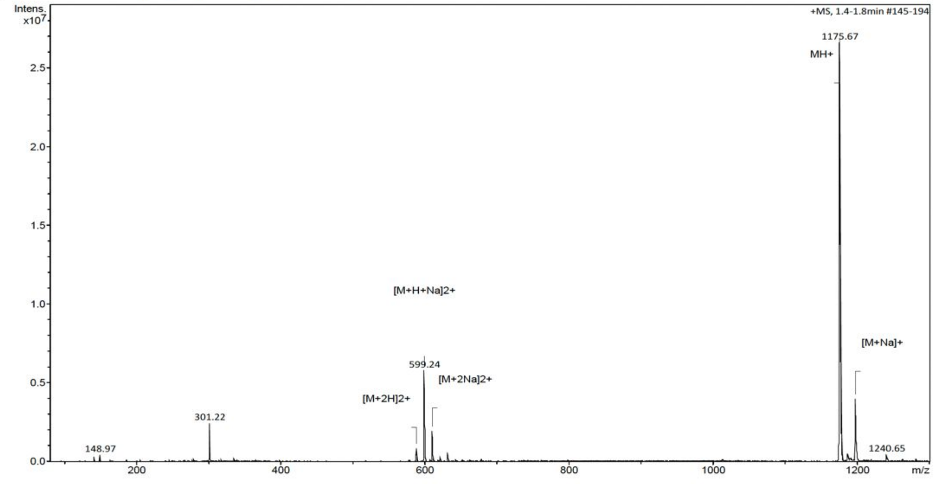

Bruker Compass DataAnalysis 4.2 printed: 6/3/2021 10:56:25 AM by: KK Page 1 of 1

Figure S1e

A6 (glucoside) 2LS186-3

Sequence: Tyr-c-[*D*-Lys-1-Nal-Phe-Glu]-Ser(ß-Glc)-CONH$_2$

Structure:

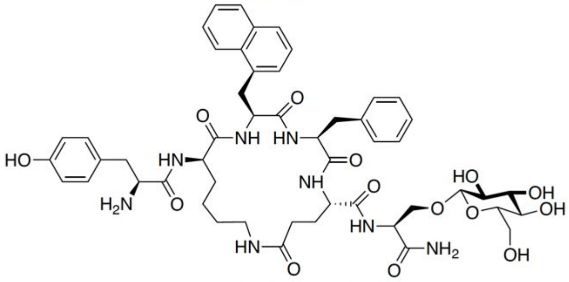

Chemical Formula: $C_{51}H_{64}N_8O_{14}$
Exact Mass: 1012.45420
Molecular Weight: 1013.11500

Mw: **1127.1** (as TFA salt)

Solubility: H_2O, Saline

Storage Condition: Dry condition, < 5°C

HPLC:

Data File:	c:\star\data\2laj\2ls186\2ls186-final-pure'	Operator (Calc):	polt lab
Channel:	2 = 280.00 nm RESULTS	Calc Date:	05/28/2021 02:47:56
Sample ID:	2ls186-3	Times Calculated:	4
Operator (Inj):	polt lab	Calculation Method:	c:\docume~1\poltlab\locals~1\temp\~2ls1
Injection Date:	05/28/2021 01:50:25 PM	Instrument (Calc):	varian
Injection Method:	c:\star\method\lajos\lsz_2_analit.mth	Run Mode:	Analysis
Run Time (min):	23.013	Peak Measurement:	Peak Area
Workstation:		Calculation Type:	Percent
Instrument (Inj):	varian	Calibration Level:	N/A
		Verification Tolerance:	N/A

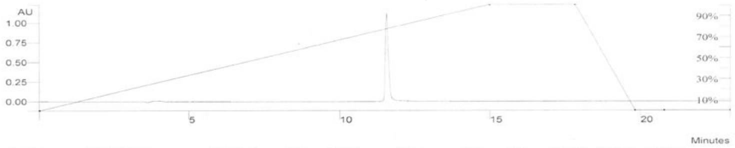

Peak No	Peak Name	Result ()	Ret. Time (min)	Time Offset (min)	Area (counts)	Rel Ret Time	Sep. Code	Width 1/2 (sec)	Status Codes	Group
1		100.0000	11.573	0.000	43291616	0.00	BB	6.7		0
	Totals	**100.0000**		**0.000**	**43291616**					

Mass spectra:

Generic Display Report

**Analysis Info**		Acquisition Date	6/3/2021 11:03:57 AM
Analysis Name	D:\Data\2021_06\LZS-4229269\4229269_2ls186-3_00.d		
Method	Tuning Mix.m	Operator	KK
Sample Name	4229269_2ls186-3_	Instrument	amaZon SL
Comment	dissolved in ACN, diluted 10x in ACN/H20		

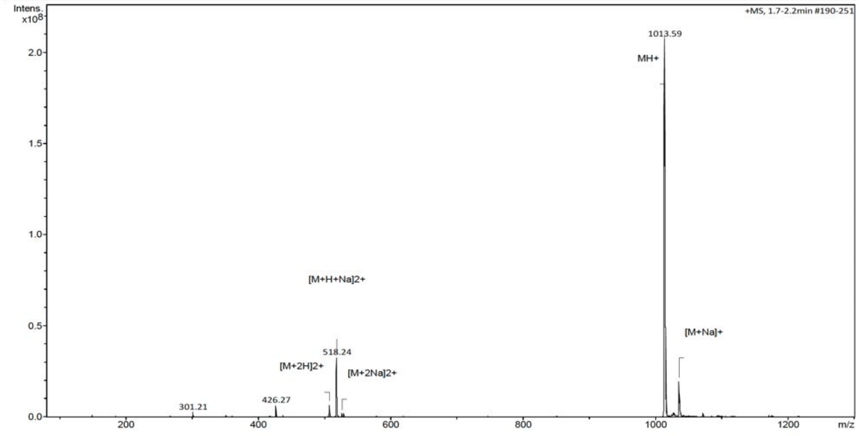

Bruker Compass DataAnalysis 4.2 printed: 6/3/2021 11:09:30 AM by: KK Page 1 of 1

Figure S1f

A7 (double glucoside) 2LS186-4

Sequence: Tyr-c-[*D*-Lys-Trp-Phe-Glu]-Ser(ß-Glc)-CONH$_2$

Structure:

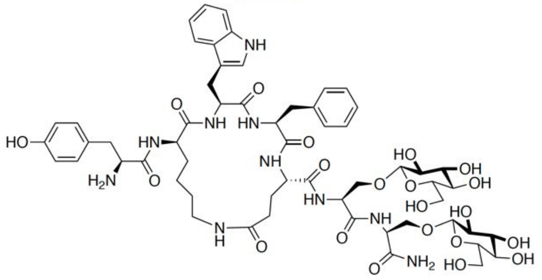

Chemical Formula: $C_{58}H_{78}N_{10}O_{21}$
Exact Mass: 1250.53430
Molecular Weight: 1251.3

Mw: **1365.3** (as TFA salt)

Solubility: H_2O, Saline

Storage Condition: Dry condition, < 5°C

HPLC:

Data File:	c:\star\data\2laj\2ls186\2ls186-final-pure'	Operator (Calc):	polt lab
Channel:	2 = 280.00 nm RESULTS	Calc Date:	05/28/2021 02:46:30
Sample ID:	2ls186-4	Times Calculated:	2
Operator (Inj):	polt lab	Calculation Method:	c:\docume~1\poltlab\locals~1\temp\~2ls1
Injection Date:	05/28/2021 02:16:45 PM	Instrument (Calc):	varian
Injection Method:	c:\star\method\lajos\lsz_2_analit.mth	Run Mode:	Analysis
Run Time (min):	23.013	Peak Measurement:	Peak Area
Workstation:		Calculation Type:	Percent
Instrument (Inj):	varian	Calibration Level:	N/A
		Verification Tolerance:	N/A

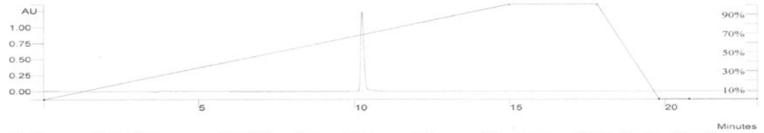

Peak No	Peak Name	Result ()	Ret. Time (min)	Time Offset (min)	Area (counts)	Rel Ret Time	Sep. Code	Width 1/2 (sec)	Status Codes	Group
1		100.0000	10.251	0.000	46398316	0.00	BB	6.7		0
	**Totals**	**100.0000**		**0.000**	**46398316**					

Mass spectra:

Generic Display Report

**Analysis Info**		Acquisition Date	6/3/2021 11:16:35 AM
Analysis Name	D:\Data\2021_06\LZS-4229269\4229269_2ls186-4_00.d		
Method	Tuning Mix.m	Operator	KK
Sample Name	4229269_2ls186-4_	Instrument	amaZon SL
Comment	dissolved in MeOH, diluted 10x in ACN/H20		

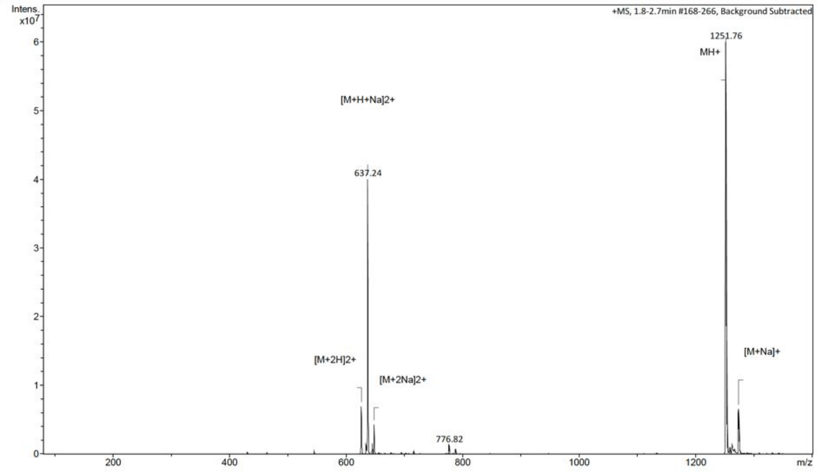

Bruker Compass DataAnalysis 4.2 printed: 6/3/2021 11:27:28 AM by: KK Page 1 of 1

Figure S1g

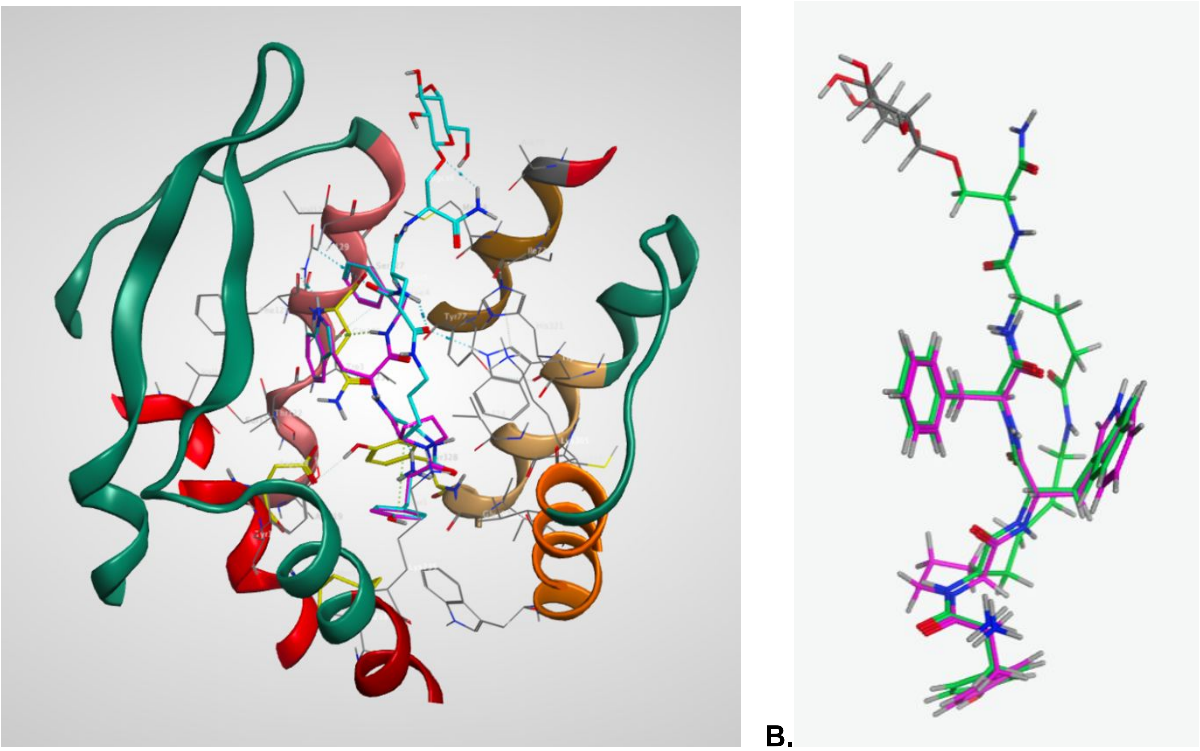

A. B.

FIGURE S2. ***In Silico* Modeling of EM1 and A1 in MOR**. **A**. Endomorphin (EM1-colored pink) and **A1** (colored cyan) interactions are overlaid within the orthosteric binding cavity of MOR (from PDB:8F7R). Specific interactions of amino acids of the MOR receptor are colored yellow in stick forms. Main transmembrane helices with specific interactions to amino acids are visible: Gln123 of TMH-II, Asp149 and Met153 of TMH-III and Tyr328 of TMH-VII. Only the polar hydrogens are shown to enhance visibility. **B**. Minimized (AMBER10) structures of **EM1** and **A1** are overlaid in the absence of the MOR receptor.

The protein structure of 8F7R[3] with **EM1** bound into the active site was processed by MOE® (https://www.chemcomp.com/index.htm) for clean-up and 3D protonation. **EM1** was selected while still bound in the cavity and its interactions with the orthosteric binding pocket using ligand interactions as articulated by MOE. The endogenous **EM1** peptide ligand was then selected and

duplicated using ligand duplicate option. One of the duplicate copies was labeled and selected for further modifications to generate targeted **A1** structure *in silico*. The Protein Builder module of MOE® was used to mutate Pro^3 to $D\text{-}Lys^3$ followed by Phe^4 extension into Glu^5 and beta-D-Gluc-Ser^6-NH_2. The modified ligand was selected, and energy-minimized in the presence of **EM1**. The lactam bridge between $D\text{-}Lys^3$-and Glu^5-gamma-COOH was built using Builder in MOE®. Then the lactam group was selected and minimized and examined for its integrity. The modified structure was then labeled as **A1** and the alignment between the two structures **EM1** and glucoside **A1** optimized by mild flexible alignment using rigid body option.

The pair of structures **EM1** and **A1** were examined for their interactions with the MOR cavity (**Figure S2**) and it was found that all specific amino acids of the receptor aligned to interact with the specific amino acids of **A1** and **EM1**. It was noticed that the sugar moiety of **A1** was extended toward the EC2 and EC3 loops, which likely interferes with binding to the MOR receptor compared **EM1**, which is consistent with the experimental binding data.

***In vivo* Methods**

Blood Brain Barrier Penetration and Pharmacokinetics

Animals: Male, Sprague-Dawley rats (225-325, *n* = 3/group; Envigo RMC Inc., Indianapolis, IN were used for *in vivo* microdialysis and pharmacokinetic experiments. Rats were co-housed in a temperature/humidity-controlled room on a 12-h reversed light/dark cycle. Food and water were available ad libitum. Animal studies were approved by The University of Arizona Institutional Animal Care and Use Committee, in accordance with NIH's Care and Use of Laboratory Animals guidelines.

Carotid Artery Catheterization and Microdialysis Surgery: Both procedures were performed in the same anesthetic event in accordance with our previous studies, with one exception, the left carotid artery was catheterized in each animal.[2]

***In Vivo* Microdialysis and Plasma Sampling:** Blood-brain-barrier penetration and pharmacokinetics of the **A1** glucoside and ZH853 (n=3/group) were determined in each rat at the same time to minimize injection site variations and individual differences between the animals. Sampling of brain dialysate and blood was conducted as previously demonstrated and with the following changes (**Figure S3**).[2, 4] The microdialysis probe (FZ-8-04; Amuza, San Diego, CA) was inserted into the striatum and perfused with aCSF (15 mM Tris HCl, 10 mM Tris base, 126 mM NaCl, 2.5 KCl, 1.2 mM NaH_2PO_4, 2mM Na_2SO_4, and adjusted pH to 7.4) at a rate of 0.5 μL/minute using one channel of a dual syringe pump (Fusion 101A Syringe Pump; Chemyx, Stafford, TX). **A1** glucoside (10 mg/kg) and **ZH853** were diluted in 0.9% USP grade sterile saline (VetOne, Boise, ID) and injected via the lateral tail vein. Brain dialysate samples used to estimate CSF were collected over 10 minutes, where t = 0 corresponds to the injection: t = -10, 0, 10, 20, 30, 40, 50, and 60 minutes. Blood was sampled to correspond with the median timepoint of the dialysate fraction, where t = 0 corresponds to the injection: t = -5, 0, 1, 5, 15, 25, 35, 45, and 55

minutes. To separate plasma from whole blood, each sample was collected in ethylenediamine tetraacetic acid capillary tubes (CT-CB300-K2EDTA; Sarstedt, Nümbrecht, Germany), inverted repeatedly for 30 seconds, and centrifuged for 10 minutes at 2,000 x g in a refrigerated centrifuge (Galaxy Mini Centrifuge; VWR, Radnor, PA).

Bioanalytical Method for the Detection of A1 (glucoside) in Brain Dialysate and Plasma: Brain dialysate (5 μL) and plasma (5 μL) were mixed with 5 μL and 50 μL of preservation solution (10% Acetic acid with 2% Acetonitrile and internal standard (DADLE, [D-Ala^2, D-Leu^5]-Enkephalin), respectively, and then vortexed for 10 seconds. 90 μL of cold acetone and deionized water (75:25, v/v) was added to precipitate dialysate and plasma proteins. After vortexing for 30 seconds, samples were centrifuged at 13,000 rpm for 15 minutes at 4 °C. The supernatant was evaporated using a Savant Speedvac concentrator. Dialysate and plasma samples were reconstituted in a 20 μL and 200 μL diluent (2% acetonitrile and 0.1% formic acid in deionized water), respectively. Samples were injected into the ThermoFisher UHPLC-MS/MS TSQ system. Chromatographic separation was achieved on an ACQUITY UPLC HSS T3 C18 Column (100 Å, 1.8 μm, 2.1 X 100 mm) and VanGuard pre-column (HSS T3 1.8 μm, 2.1 X 5 mm) with the temperature set at 20 ℃. A water-acetonitrile gradient with a 0.3 mL/minute flow rate was used. The mobile phases used were A: 0.1% v/v formic acid in deionized water and B: 0.1% v/v formic acid in acetonitrile. An injection volume of 8 μL was used. Detection was achieved using a Thermo Fisher TSQ Fortis Plus mass spectrometry instrument with an H-ESI ion source. The instrument was operated in a positive ion mode. The MRM mode was used for quantification using target ions at m/z 501.92 → 420.83 (quantifier), 736.33 (qualifier) for doubly protonated **A1** glucoside (MW 1003.1 gmol^{-1}); Target ions at m/z 405.68 → 332.01 (quantifier), 736.18 (qualifier) for doubly protonated ZH853 (MW 809.92 gmol^{-1}). Concentrations were calculated using the response factor-concentration plot. Pharmacokinetic parameters, including the area under the curve (AUC), maximum plasma concentration (C_{max}), the time to reach C_{max} (T_{max}), and half-life ($t_{1/2}$), were

determined using the PKSolver.[5] CSF concentration is converted to estimated concentration using the microdialysis probe recovery rate. All data are presented as mean ± SEM.

Following intravenous bolus injection, **A1** glucoside dose exposure was observed in the plasma with a mean AUC of 157.1 ± 36.6 μM*minute, a C_{max} value of 15.7 ± 7.4 μM. The half-life ($t_{1/2}$) was 25.9 ± 5.7 minutes, and the T_{max} was 1-minute post-injection (n = 3). For the dialysate, the mean AUC value was 141.0 ± 69.2 μM*minutes with a mean C_{max} value of 10.8 ± 6.5 μM. The $t_{1/2}$ was 16.6 ± 7.9 minutes, and T_{max} was 5 minutes (n = 3) post-dose administration. ZH853 analog dose exposure in the plasma was observed with a mean AUC of 109.7 ± 36.7 μM*minute and a C_{max} of 16.5 ± 3.0 μM. The half-life ($t_{1/2}$) was 15.9 ± 2.3 minutes, and the T_{max} was 2 minutes (n = 3). For the dialysate, the mean AUC was 20.7 ± 7.9 μM*minutes with a mean C_{max} value of 1.5 ± 0.2 μM. The $t_{1/2}$ was 6.1 ± 0.7 minutes, and T_{max} was 28 minutes (n = 3) post-dose administration.

Antinociception Behavior

Animals: Male and female CD-1 and DBA mice, 21–29 g at testing, were obtained from Charles River (Wilmington, MA) and housed in a 12-h light/dark cycle. All experiments were approved by the Tulane Institutional Animal Care and Use Committee and conducted according to the NIH Guide for the Care and Use of Laboratory Animals. All efforts were made to minimize animal suffering and to reduce the number of animals used.

Tail Flick (TF): CD1 mice were used for this test. The latency to withdraw the tail from a heat source was automatically measured (IITC, Woodland Hills, CA). Baseline latencies were 3-4 s with a cutoff time of 9 s to prevent tissue damage. Percent Maximum Possible Effect (%MPE) was determined as [(latency-baseline latency)/(9-baseline latency)]*100. Peak responses were later for the glycopeptides (40-60 min) compared to the reference compound ZH853 (30-40 min) and morphine (20-30 min). The average MPE at each of those respective times was therefore

calculated and used as the peak response for determining ED_{50}'s. Initial tests, based on previous studies with **ZH853**, were 3h in duration. As shown in **Figure 1**, however, some of the glycopeptide analog doses, particularly **A2**, produced an unexpectedly long duration of antinociception that remained well above 50% MPE at 3h. Additional subgroups (n = 4-5) were therefore tested for 5h. For **A7**, all animals were tested for 5h. For the time course (**Figure 3**) and the dose-response curve/ED_{50} calculations (**Figure 4**), which were based on < 1h values, all animals were used. Duration of antinociception was assessed using both the area under the curve (AUC) and duration of antinociception, calculated as the time from the first to the last time point greater than or equal to 50% MPE. For these two measures, because a majority of animals in the top 2 doses for lactoside **A2** and the top dose of glucoside **A1** were greater than 50% MPE at 3h, therefore underestimating the true AUC and duration, only the 5h subgroups were used in AUC and duration calculations for these groups. This method is further supported as conservative since average scores remained above 50% MPE in the top doses of **A2** even at the 5h time point as shown in **Figures 2** and **3**.

Hot Plate (HP) Test: This test reflects a supraspinally organized complex response,[6] and was used to assess the CNS activation of antinociception by peripherally (s.c.) administered compounds. In preliminary studies, we found that DBA mice showed HP responses at lower doses of all compounds tested, including morphine, than CD1 mice and were therefore used for this test to reduce test compound requirements. The HP apparatus (IITC, Woodland Hills, CA) was set to 55.5°C, a temperature that elicited a response after 7-9 sec. Three baseline HP latencies to rapidly lift, lick, or shake the hind paws were taken prior to drug injection. Mice were removed from the HP after a maximum of 30 sec. Mice were injected with several analogs (0–5.6 mg/kg s.c.) and tested from 30–300 min after injection. Data were converted to maximum possible effect (%MPE) as described for TF. Peak responses were later for the glycopeptides (45-60 min) compared to the reference compounds **ZH853** and morphine (30-45 min). The average MPE at

each of those respective times was therefore calculated and used as the peak response for determining ED_{50}'s. These values were used to calculate area under the curve (AUC). The duration of antinociception greater than 50% MPE was calculated as with TF.

No unexpected or unusually high safety hazards were encountered for the methods used here.

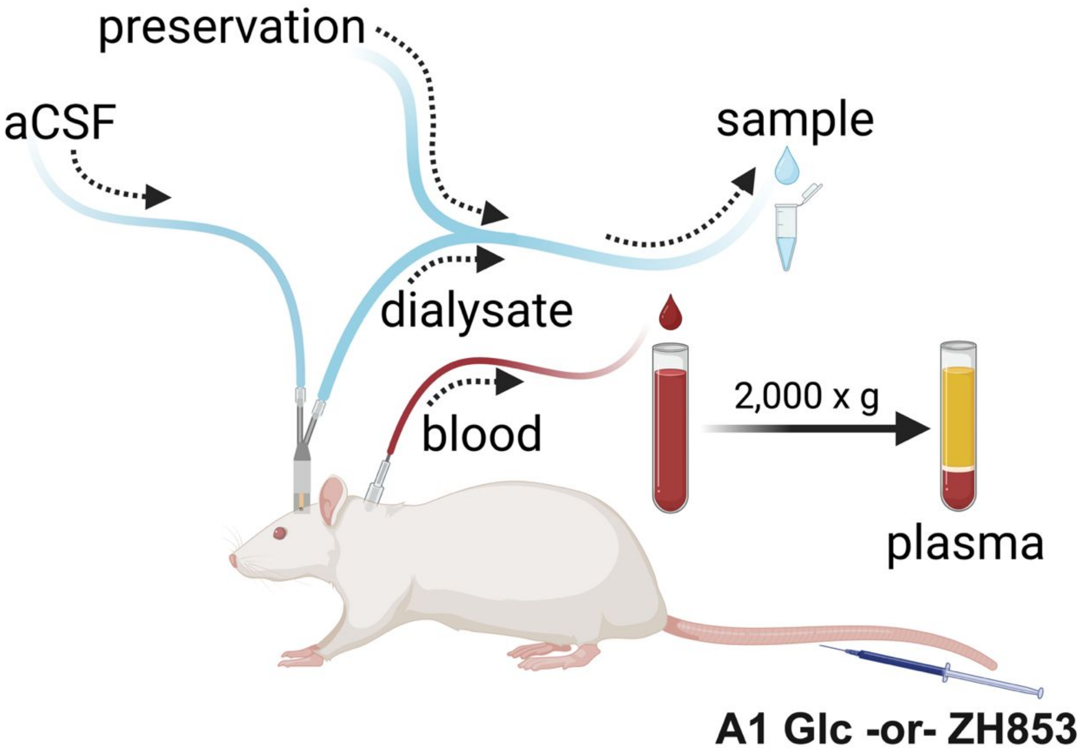

Figure S3. Microdialysis and Pharmacokinetics Experiment Scheme.

Scheme depicts catheterization and microdialysis of the rat striatum. **A1** Glc = **A1** glucoside endomorphin-1 analog (10 mg/kg; *i.v.*) **ZH853** = **ZH853**•HCl (10 mg/kg; *i.v.*); aCSF = artificial cerebrospinal fluid. Created with BioRender.com (Agreement number MH26RXD2YP).

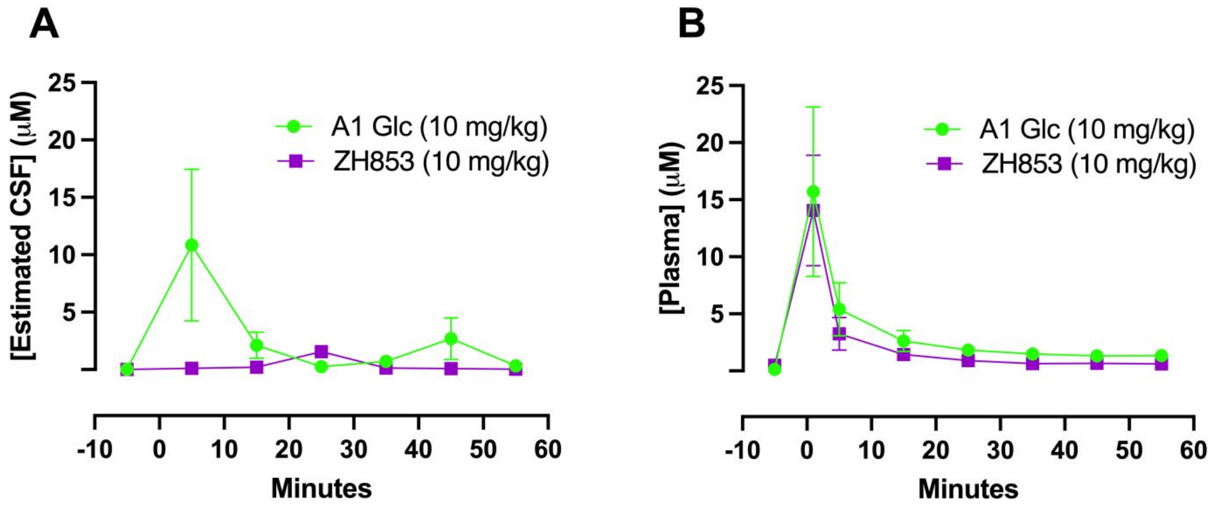

Figure S4. Time course of *In Vivo* Blood Brain Barrier Penetration and Plasma Pharmacokinetics. A1 (Glucoside) and **ZH853** were injected (10 mg/kg, *i.v.*) into the lateral tail vein. (A) Time course of estimated CSF (brain dialysate) concentration (mean ± SEM) of **A1** (n=3) compared to **ZH853** (n=3) in aCSF from 0-60 minutes. (B) Time course of plasma concentration (mean ± SEM) of **A1** Glc (n=3) compared to **ZH853** (n=3) from 0-55 minutes.

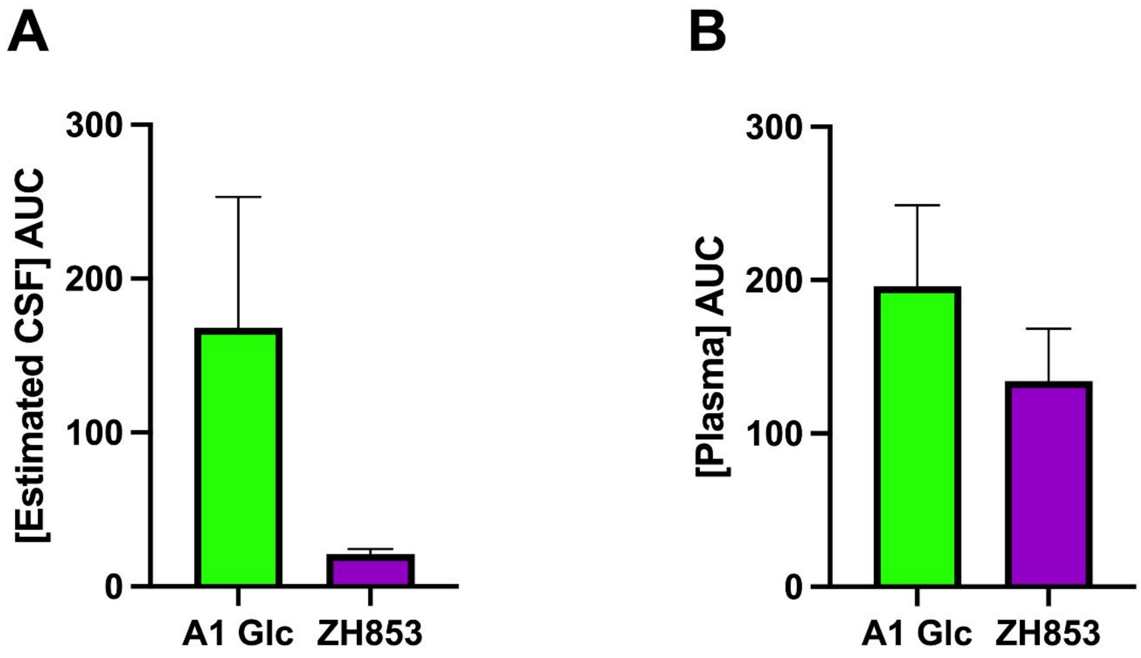

Figure S5. Area Under the Curve (AUC) of *In Vivo* Blood Brain Barrier Penetration and Plasma Pharmacokinetics. **A1** (Glucoside) and **ZH853** were injected (10 mg/kg, *i.v.*) into the lateral tail vein. AUCs (mean ± SEM) were obtained by integrating the pharmacokinetic profile over time for the estimated CSF (brain dialysate; 0-60 minutes) and plasma concentrations (0-55 minutes). Additional time for estimated CSF is due to each timepoint representing the median value collected over 10 minutes. (A) AUC of estimated CSF (brain dialysate) concentrations of **A1** (n=3) compared to **ZH853** (n=3) in aCSF from 0-60 minutes. **A1** showed an ~8-fold increase over **ZH853** in the CSF. (B) AUC of plasma concentrations of **A1** (n=3) compared to **ZH853** (n=3) in aCSF from 0-55 minutes. **A1** showed an ~1.5-fold increase over **ZH853** in the plasma.

References

(1) Apostol, C. R.; Hay, M.; Polt, R. Glycopeptide drugs: a pharmacological dimension between "Small Molecules" and "Biologics". *Peptides* 2020, *131*, 170369. DOI: 10.1016/j.peptides.2020.170369.

(2) Szabo, L. Z.; Tanguturi, P.; Goodman, H. J.; Sprober, S.; Liu, C.; Al-Obeidi, F.; Bartlett, M. J.; Falk, T.; Kumirov, V. K.; Heien, M. L.; et al. Structure-based design of glycosylated oxytocin analogues with improved selectivity and antinociceptive activity. *ACS Med. Chem. Lett.* 2023, *14* (2), 163-170. DOI: 10.1021/acsmedchemlett.2c00455.

(3) Wang, Y.; Zhuang, Y.; DiBerto, J. F.; Zhou, X. E.; Schmitz, G. P.; Yuan, Q.; Jain, M. K.; Liu, W.; Melcher, K.; Jiang, Y.; et al. Structures of the entire human opioid receptor family. *Cell* 2023, *186* (2), 413-427 e417. DOI: 10.1016/j.cell.2022.12.026.

(4) Hay, M.; Polt, R.; Heien, M. L.; Vanderah, T. W.; Largent-Milnes, T. M.; Rodgers, K.; Falk, T.; Bartlett, M. J.; Doyle, K. P.; Konhilas, J. P. A novel angiotensin-(1-7) glycosylated Mas receptor agonist for treating vascular cognitive impairment and inflammation-related memory dysfunction. *J. Pharmacol. Exp. Ther.* 2019, *369* (1), 9-25. DOI: 10.1124/jpet.118.254854.

(5) Zhang, Y.; Huo, M.; Zhou, J.; Xie, S. PKSolver: an add-in program for pharmacokinetic and pharmacodynamic data analysis in Microsoft Excel. *Comput. Methods Programs Biomed.* 2010, *99* (3), 306-314. DOI: 10.1016/j.cmpb.2010.01.007.

(6) Chapman, C. R.; Casey, K. L.; Dubner, R.; Foley, K. M.; Gracely, R. H.; Reading, A. E. Pain measurement: an overview. *Pain* 1985, *22* (1), 1-31.
